# Exosomes in ovarian cancer: Impact on drug resistance and advances in SERS detection techniques

**DOI:** 10.1016/j.jpha.2024.101170

**Published:** 2024-12-18

**Authors:** Biqing Chen, Xiaohong Qiu, Yang Li

**Affiliations:** a Gynaecology and Obstetrics, The Second Affiliated Hospital of Harbin Medical University, Harbin Medical University, Harbin, 150081, China; b College of Pharmacy, Research Center for Innovative Technology of Pharmaceutical Analysis, Harbin Medical University, Harbin, 150081, China

**Keywords:** Exosomes, Ovarian cancer, Chemotherapy resistance, Extracellular vesicles, Surface-enhanced Raman scattering (SERS)

## Abstract

Ovarian cancer is a prevalent gynecological malignancy with high mortality and low survival rates. The absence of specific symptoms in early stages often leads to late-stage diagnoses. Standard treatment typically includes surgery followed by platinum and paclitaxel chemotherapy. Exosomes, nanoscale vesicles released by various cell types, are key in intercellular communication, carrying biologically active molecules like proteins, lipids, enzymes, mRNA, and miRNAs. They are involved in tumor microenvironment remodeling, angiogenesis, metastasis, and chemoresistance in ovarian cancer. Emerging research highlights exosomes as drug carriers and therapeutic targets to suppress anti-tumor immune responses. Surface-enhanced Raman scattering (SERS) enables multiplexed, sensitive, and rapid detection of exosome surface proteins, offering advantages such as low background noise, no photobleaching, robustness, and high sensitivity over other detection methods. This review explores the relationship between exosomes and chemoresistance in ovarian cancer, examining the mechanisms by which exosomes contribute to drug resistance and their clinical implications. The goal is to provide new insights into chemoresistance mechanisms, improve diagnosis and intervention strategies, and enhance chemotherapy sensitivity in clinical treatments. In addition, the prospects of exosomes as drug carriers to resist chemical resistance and improve the survival of ovarian cancer patients are summarized. This article emphasizes the role of SERS in detecting ovarian cancer exosomes and advances in exosome detection.

## Highlights

• Chemotherapy failure and drug resistance are clinical challenges in patients with ovarian cancer.
• Ovarian cancer can induce chemoresistance through exosomes.
• Exosomes are also potential delivery tools for the treatment of ovarian cancer.
• The therapeutic potential of exosomes in improving the prognosis of ovarian cancer patients.

## Graphical abstract

This review provides a comprehensive overview of the utilization of exosomes in the context of ovarian cancer, examining their role in biogenesis, biological structure, biological function, chemotherapy resistance, and their potential limitations as drug delivery vehicles.

**Figure:**
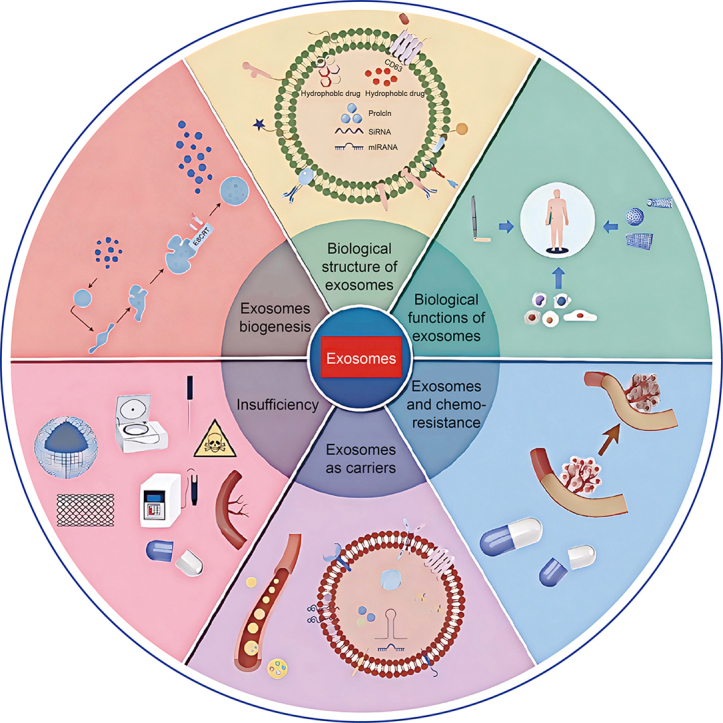

## 1 Introduction

Ovarian cancer is the deadliest gynecologic malignancy and ranks as the eighth most common cause of cancer-related mortality among women globally [1]. The standard treatment for ovarian cancer includes initial surgery followed by adjuvant chemotherapy with platinum and taxane agents. For patients with advanced disease, the approach involves cytoreductive surgery to reduce tumor burden, followed by combination chemotherapy. The advanced stage at diagnosis is often due to the lack of specific clinical symptoms in the early stages. Despite an initial favorable response to platinum-based chemotherapy, many patients relapse due to the development of chemoresistance, which poses a significant challenge in treating ovarian cancer. Approximately 70% of patients experience recurrence, and the 5-year survival rate remains only around 30% [2]. The mechanisms underlying chemotherapy resistance include increased glutathione synthesis, enhanced DNA repair, altered drug efflux and influx, drug inactivation, modifications in drug targets, suppression of apoptotic pathways, activation of the epithelial-mesenchymal transition (EMT) pathway, involvement of miRNA-mediated mechanisms, and promotion of angiogenesis [[3], [4], [5]]. These factors highlight the need for effective strategies to overcome drug resistance [6,7].

Extracellular vesicles (EVs) are membrane-bound structures ubiquitously produced by mammalian cells [8,9]. Recent studies emphasize their role in intercellular communication within the tumor microenvironment, with exosomes, a subset of EVs derived from endosomes, being particularly significant. Exosomes carry diverse molecular contents, including DNA, RNA, proteins, and lipids, influencing ovarian cancer progression and metastasis through mechanisms like EMT, miRNA regulation, angiogenesis, immunosuppression, and premetastatic niche formation [10,11]. They also play a crucial role in immune evasion by inhibiting the surveillance activities of various immune cells, such as macrophages, dendritic cells, natural killer (NK) cells, B cells, T cells, and myeloid-derived suppressor cells (MDSCs) [12]. Furthermore, exosomes from ovarian tumor cells can manipulate the extracellular matrix, induce EMT, enhance cancer cell migration, and facilitate premetastatic niche formation, allowing transendothelial migration of cancer cells into the bloodstream [[13], [14], [15]]. Exosomal surface particles regulate cell migration pathways and contribute to the development of ovarian cancer stem cells [16]. These exosomes can initiate chemoresistance in advanced stages of ovarian cancer and potentially serve as delivery vehicles for therapy and platforms for enhancing immune responses against cancer [[17], [18], [19]].

Traditional methods for detecting circulating tumor-secreted exosomes, such as enzyme-linked immunosorbent assay (ELISA), often involve large sample volumes, complex sample preparation, and time-consuming processes. Currently, the primarily reported methods for exosome detection are based on immune recognition, specifically using antibodies to capture exosomes by targeting specific surface proteins. However, the limitations of antibodies, such as poor tolerance, difficulties in synthesis, high costs, and limited availability of immune proteins related to target proteins, hinder their further application [20]. In contrast, aptamers possess unique characteristics such as high affinity and specificity, good thermochemical stability, and ease of synthesis and modification, which present significant potential to replace antibody-based immunoassays. The multivalent aptamer approach can enhance binding affinity to targets by linking multiple aptamers [21]. Recently, new optical methods for exosome detection have been reported, achieving significant advances in simplifying detection protocols and improving sensitivity. Raman spectroscopy is a non-invasive technique that produces the fingerprint vibration spectrum of analytes upon laser irradiation. When introducing plasmonic metal nanomaterials, surface-enhanced Raman scattering (SERS) can amplify the Raman signal by up to 10^14^–10^15^, providing high sensitivity for target analyses. SERS is a powerful analytical tool that can significantly enhance the Raman scattering of molecules adsorbed on plasmonic nanostructures [22]. The enhancement effect of SERS primarily arises from both chemical and electromagnetic field amplification, where metallic nanoparticles play a unique role in amplifying the Raman signals. SERS has high sensitivity in describing molecular vibration modes [23], and its label-free, non-invasive advantages offer great feasibility for biosensing and therapeutic applications. SERS is a powerful method for identifying chemical information at the single-molecule scale [24], exhibiting high sensitivity and specificity.

In recent years, SERS has found extensive applications in biochemical analysis, including pesticide residue detection [25], virus detection [26,27], tumor identification [28], drug safety, environmental monitoring, and more. Many researchers have employed SERS to identify tumor-derived exosomes [29], with approaches categorized into labeled methods and label-free methods [30]. Labeled methods offer high accuracy and semi-quantitative analysis but involve complex procedures [31]. These methods only glean information from the labels, potentially missing the actual exosome signals and leading to misinterpretations. To effectively screen exosomes, there is a need to develop multifunctional probes capable of simultaneously identifying different types of exosomes. Label-free methods are simpler but have poorer capabilities in distinguishing different tumor exosomes [32]. SERS can transmit unique spectral signals in complex biological environments due to its fingerprint characteristics and narrow spectral bandwidth, enabling excellent multiplexing capability and high sensitivity in multiplex detection. Given the need for early disease detection, developing methods for detecting exosomes, especially for screening different types of exosomes, remains a significant challenge [33]. For nanoscale exosomes, microfluidic chips offer advantages such as size miniaturization, portability, and precise control over small volume liquid samples, making them an ideal platform for exosome detection. Research that employs microfluidic-based analysis of ovarian cancer-derived exosomes is scarce [34]. In disease detection and treatment, time is extremely valuable. Rapid identification of disease types can save substantial therapeutic time. Currently, there is a lack of highly sensitive and accurate methods for early diagnosis of ovarian cancer. Now, we anticipate that sensitive SERS platforms for detecting ovarian cancer exosomes will enhance our understanding of diagnosis and treatment resistance in ovarian cancer patients.

The primary objective of this review is to elucidate the role of exosomes in mediating chemotherapy resistance in ovarian cancer and explore their therapeutic potential in improving patient outcomes.

## 2 Exosome biogenesis

Exosome biogenesis is a complex process involving multiple pathways that orchestrate the formation, maturation, and release of these nanoscale vesicles. The primary pathway is the endosomal sorting complex required for transport (ESCRT)-dependent mechanism, which is crucial for the creation of intraluminal vesicles (ILVs) within multivesicular bodies (MVBs) [35]. This process begins with the ESCRT-0 complex identifying and sequestering ubiquitinated proteins. Subsequently, the ESCRT-I and ESCRT-II complexes are recruited, facilitating vesicle budding and cargo sorting. The final stage involves the ESCRT-III complex, which drives membrane scission, releasing ILVs into the MVB lumen. In addition to the ESCRT machinery, exosome biogenesis can occur through ESCRT-independent pathways, which utilize lipid-mediated endocytosis [36]. In these pathways, ceramides and sphingolipids play a pivotal role, organizing into lipid raft microdomains on the plasma membrane, thereby promoting membrane curvature and the formation of ILVs without the need for ESCRT proteins. Ceramides, in particular, are known to induce membrane budding by generating spontaneous negative curvature, a key step in vesicle formation [37].

**Fig. 1:**
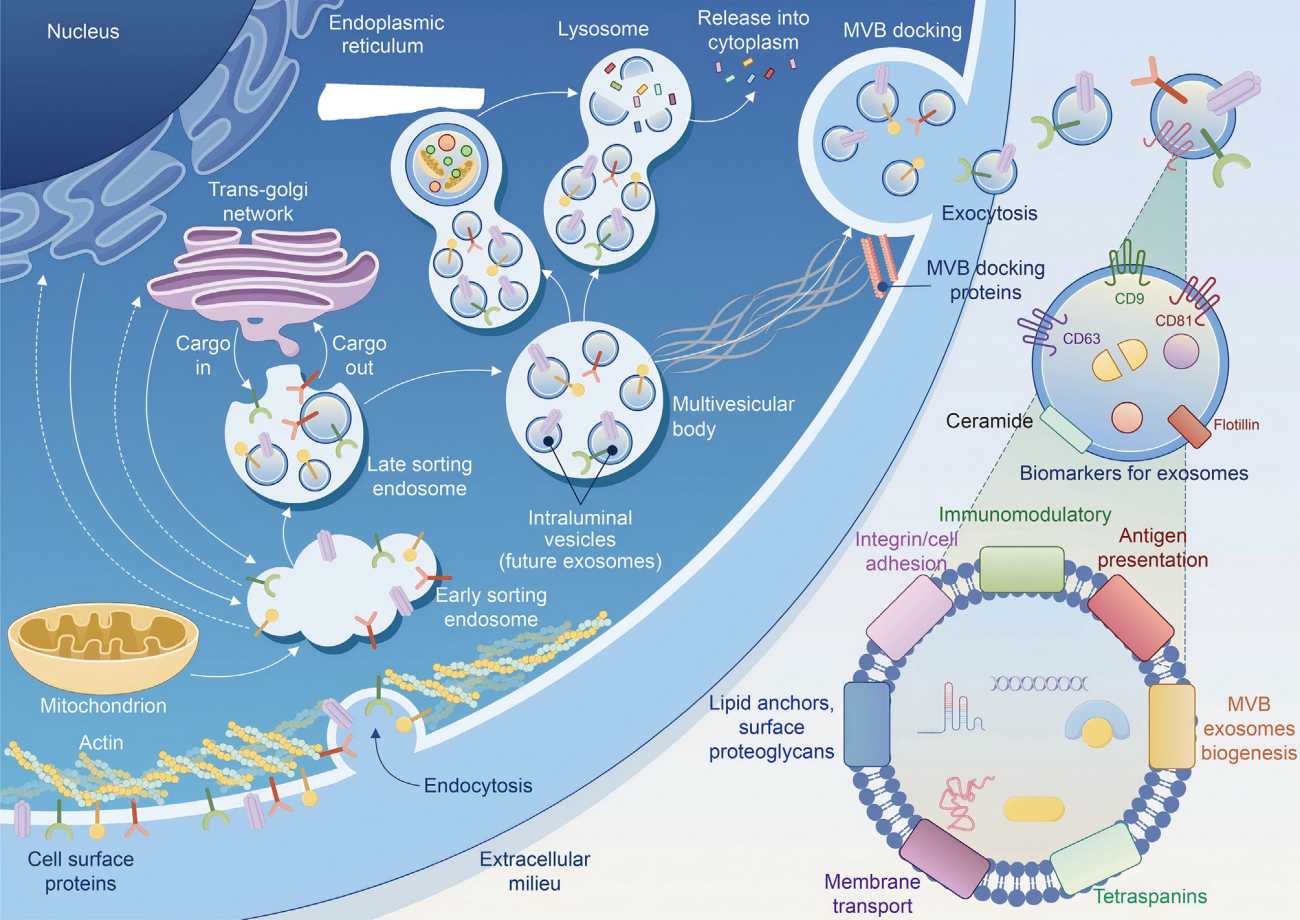
Exosome biogenesis involves multiple pathways, including the endosomal sorting complex for transport (ESCRT)-dependent pathway, lipid-mediated endocytosis, and tetraspanin-mediated mechanisms, which play key roles in cargo transport, membrane deformation, and exosome secretion. MVB: multivesicular bodies; CD9: cluster of differentiation 9.

Tetraspanin-enriched microdomains represent another critical aspect of exosome biogenesis. Tetraspanins, such as cluster of differentiation (CD9), CD63, and CD81, are integral membrane proteins that facilitate various stages of exosome formation. These proteins aid in the sorting of cargo to the MVB, organize the endosomal membrane into functional domains, and enhance the secretion of diverse molecules within exosomes [38]. The maturation and secretion of exosomes involve the progression from early endosomes, which are formed through the fusion of primary endocytic vesicles, to MVBs, characterized by the inward budding of the endosomal membrane. This inward budding sequesters cargo, resulting in the formation of ILVs [38]. MVBs can either fuse with lysosomes for degradation or merge with the plasma membrane to release ILVs as exosomes into the extracellular space. Once released, exosomes can interact with nearby cells or be transported to distant sites via the bloodstream or lymphatic system, facilitating intercellular communication. Some exosomes even possess the ability to re-fuse with the plasma membrane of the originating cell, functioning as signaling hubs [39]. The release of exosomes is tightly regulated by various molecules, including ras-related in brain guanosine triphosphatases (Rab GTPases) such as Rab27A and Rab27B, which direct MVBs to the cell periphery [40]. Additionally, high concentrations of ceramide and the involvement of soluble N-ethylmaleimide-sensitive factor attachment protein receptor (SNARE) complexes are crucial for the fusion of MVBs with the plasma membrane, culminating in the exocytosis of exosomes. The ESCRT machinery’s role concludes with the dissociation of ESCRT-III from the MVB membrane, facilitated by the sorting protein vacuolar protein sorting 4 (Vps4), which uses energy to disassemble the complex and recycle its components [41] (Fig. 1).

## 3 Biological structure of exosomes

Exosomes, ranging from 50 to 100 nm in size, are nanoscale vesicles enveloped by a lipid bilayer [11]. Exosomes are characterized by a unique double-layered spherical membrane, composed of lipid constituents such as sphingomyelin, cholesterol, and ceramide. The exosomal membrane also contains various proteins involved in transmembrane transport, including Rab GTPases, annexin, flotillin, and components of the ESCRT complex like ALG-2-interacting protein X (Alix) and tumor susceptibility gene 101 (TSG101). Heat shock proteins (HSPs), integrins, and members of the tetraspanin family, such as CD9, CD63, CD81, and CD82, are commonly found on exosomal surfaces, contributing to their stability and functionality in intercellular communication [42].

**Fig. 2:**
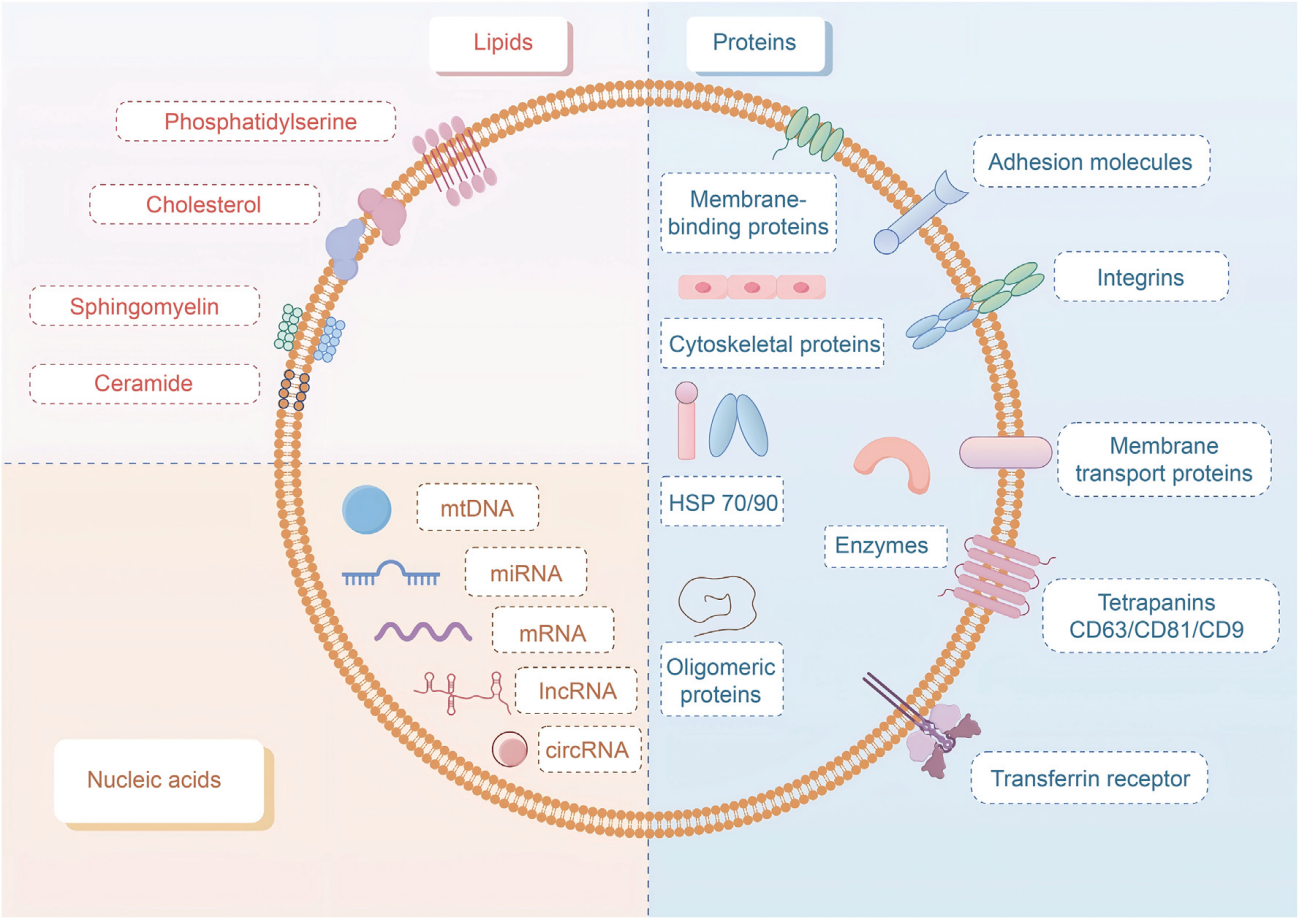
Exosomes contain diverse biomolecules essential for intercellular communication. The diagram illustrates the fundamental composition of exosomes, including the drug payload, transmembrane proteins, and various biomolecules such as proteins, DNA, and RNA, as well as receptor proteins. These components play a crucial role in facilitating intercellular communication. HSP: heat shock protein; CD63: cluster of differentiation 63.

Initially, exosomes were thought to primarily function as cellular waste disposal units, facilitating the removal of unwanted materials. However, advances in biochemical and proteomic analyses have revealed that exosomes carry specific protein and lipid signatures, indicating their active role in intercellular communication [42]. Exosomes are now recognized as important mediators in the exchange of molecular information between cells, carrying a distinct set of proteins, including those involved in plasma membrane assembly, endocytic pathways, and various cytosolic proteins. These include tetraspanins like CD9, CD36, and CD31, as well as ESCRT components such as Alix, ADP-ribosylation factor 6 (ARF6), and TSG101 [43]. Moreover, molecular biology techniques have shown that exosomes also contain genetic material, such as DNA, messenger RNA (mRNA) and microRNA (miRNA). The transfer of these nucleic acids can significantly influence gene expression in recipient cells. *In vitro* studies have demonstrated that the mRNAs contained within exosomes can be translated into proteins in target cells, highlighting their potential in modulating cellular functions [44] (Fig. 2).

## 4 Biological functions of exosomes

Exosomes play a crucial role in intercellular communication by transporting various molecules from tumor-initiating cells. These molecules include proteins such as major histocompatibility complex (MHC) Class I and II, which are involved in antigen presentation, annexins and Rab proteins, which regulate membrane-cytoskeleton dynamics and membrane fusion, and signal transduction proteins like G proteins [[45], [46], [47]]. Exosomal lipids are also essential for maintaining the structural integrity and functionality of exosomes, influencing their transport, architecture, release, and signaling capabilities. Tumor-derived exosomes are significant in modulating the tumor microenvironment, which consists of tumor cells, stromal cells, infiltrating immune cells, and cytokines. They activate extracellular receptor signaling pathways, influencing processes such as signal transduction, tumor metastasis and invasion, immune regulation (including antigen presentation and immune suppression), cell cycle distribution, apoptosis, and chemotherapy resistance through the release of bioactive molecules [48,49].

Exosomes are secreted by various cell types, including red blood cells, lymphocytes, platelets, dendritic cells, and tumor cells [50]. They are present in a wide range of biological fluids, such as plasma, saliva, nasal secretions, cerebrospinal fluid, urine, and ascites, highlighting their involvement in numerous physiological functions. These functions include immune response modulation, tissue regeneration, stem cell homeostasis, and communication with the central nervous system. Additionally, exosomes are implicated in several pathological conditions, such as cardiovascular diseases, neurodegenerative disorders, cancer, and inflammation. In the context of ovarian cancer, exosomes are crucial in mediating communication between tumor cells and their surrounding environment, affecting early detection, prognosis, chemotherapy resistance, and the efficacy of targeted therapies [51]. Identifying specific exosomal biomarkers can help elucidate the mechanisms underlying ovarian cancer progression and aid in developing innovative therapeutic interventions.

**Fig. 3:**
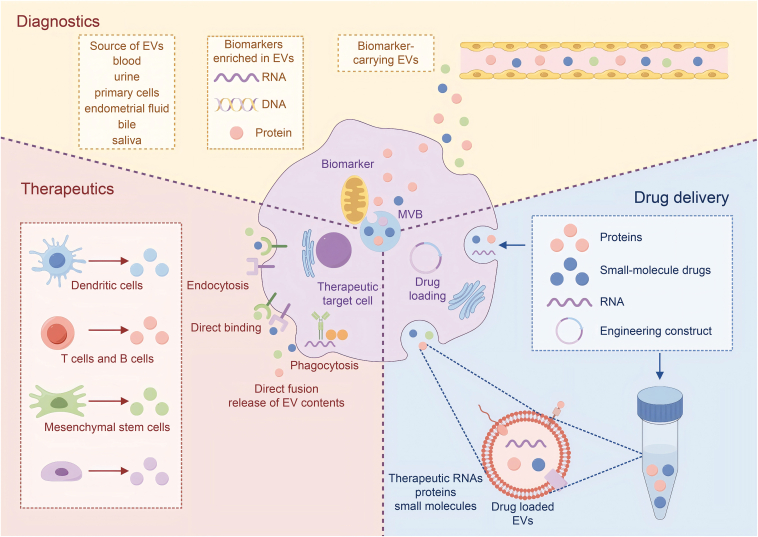
Exosomes in biological fluids serve as biomarkers and modulate physiological processes. Exosomes have been identified in numerous biological fluids, suggesting their diverse physiological effects and potential utility as biomarkers. These extracellular vesicles play a role in immune system modulation and alteration of the tumor microenvironment, as well as serving as carriers for drug delivery. EVs: extracellular vesicles; MVB: multivesicular bodies.

Exosomes serve as vital signaling entities, mediating interactions between tumor cells and other cells in the tumor microenvironment. They can carry oncogenic signals that promote tumor growth, metastasis, and resistance to therapy. The transfer of oncogenes, tumor suppressor genes, and regulatory RNAs via exosomes can alter the behavior of recipient cells, contributing to tumor heterogeneity and therapy resistance. Consequently, understanding the role of exosomes in cancer biology is essential for developing novel diagnostic and therapeutic strategies. As diagnostic biomarkers, exosomes can provide insights into the molecular characteristics of tumors, aiding in early detection and the assessment of disease progression. Furthermore, as therapeutic targets or delivery vehicles, exosomes offer promising potential for delivering drugs, nucleic acids, and other therapeutic agents directly to tumor cells, thereby enhancing treatment efficacy and reducing side effects [50] (Fig. 3).

The secretion of exosomes and their contents varies depending on the cell type, anatomical location, and physiological conditions. The composition of exosomes is highly dynamic, reflecting the state of the parent cell. This variability in content and function underscores the importance of exosomes in physiological processes, including immune response modulation, tissue regeneration, and maintenance of stem cell homeostasis. Moreover, exosomes play a role in pathological conditions such as cardiovascular and neurodegenerative diseases, cancer, and inflammation. They serve as essential signaling molecules, facilitating communication between tumor cells and the surrounding stromal and immune cells. In the context of ovarian cancer, exosomes are implicated in early detection, prognosis, chemotherapy resistance, and targeted therapy, acting as diagnostic biomarkers and therapeutic targets. The comprehensive understanding of exosomal biology and their functional roles offers valuable insights into their potential applications in medical research and clinical practice, particularly in the fields of oncology and regenerative medicine [44].

## 5 Exosomes and chemoresistance

### 5.1 Exosome miRNA and chemoresistance

**Fig. 4:**
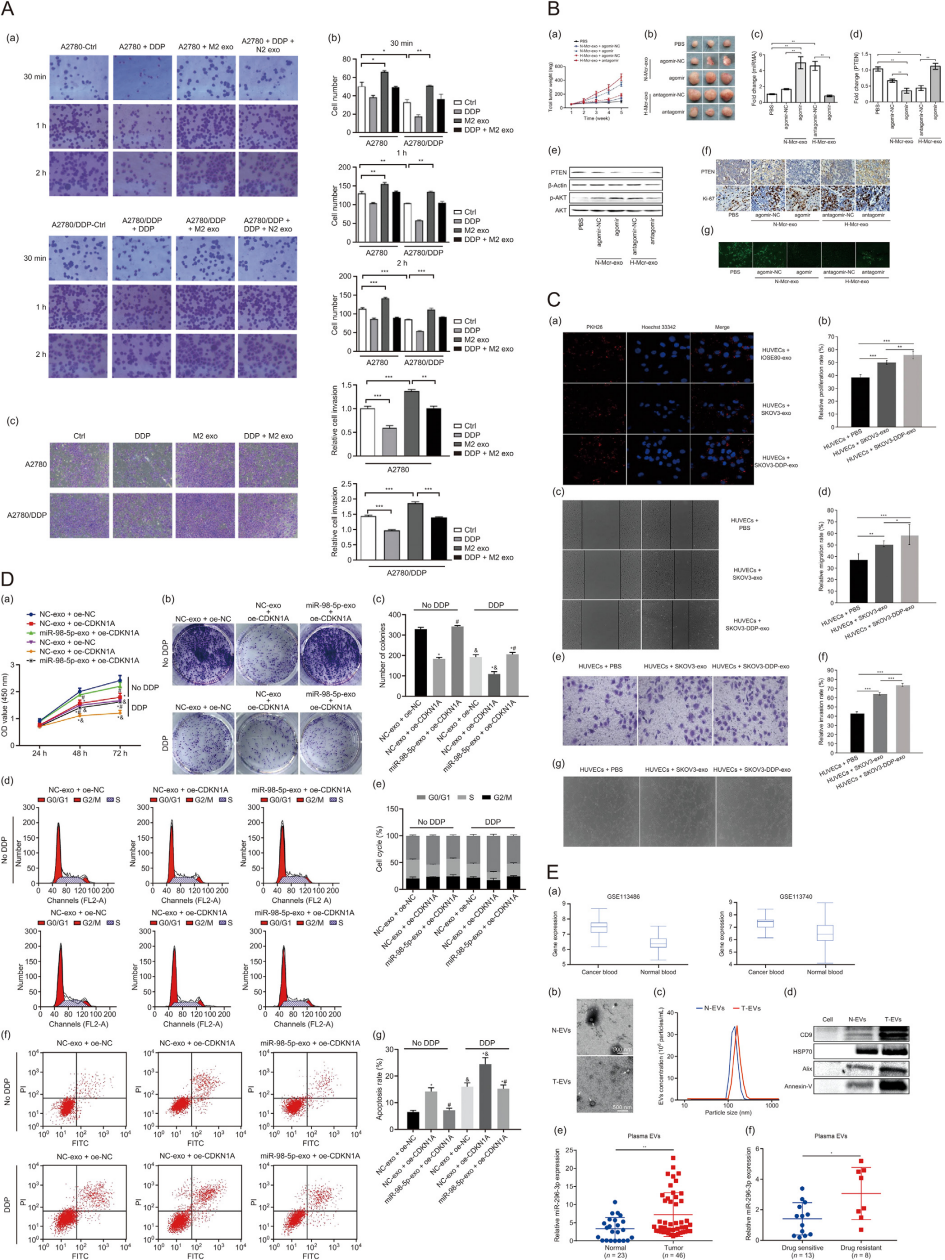
Exosomes from diverse sources drive ovarian cancer progression and chemoresistance. (A) CD163^+^ tumor-associated macrophages (TAMs) exosomes can promote the adhesion and migration of epithelial ovarian cancer (EOC) cells. (a, b) After the addition of CD163^+^ TAMs (M2 exo), the adhesion ability increased, and cisplatin (DDP) administration decreased the cell adhesion. The two kinds of joint treatments had an offset effect, and the overall adhesion of A2780/DDP cells was higher than that of the parental strain. All the results were time dependent. Crystal violet staining, 200×. (c) The two kinds of joint treatments had an offset effect, and the overall adhesion of A2780/DDP cells was higher than that of the parental strain. Crystal violet staining, 400×. All the results were time dependent, and the difference was statistically significant (M2 exo 100 µg, *n* = 3). ∗*P* < 0.05, ∗∗*P* < 0.01, ∗∗∗*P* < 0.001. Printed from Ref. [52] with permission. (B) Effects of exosomic miR-223 in cisplatin resistance *in vivo*. Exosomes derived from the normoxic or hypoxic macrophages which were transfected with agomir or antagomir respectively. SKOV3 cells were subcutaneously injected into BALB/c nude mice, we allowed potential tumors to grow for a week, then, cDDP (5 mg/kg) and exosomes (10 μg) or phosphate buffered saline (PBS) were injected into the center of the xenograft tumors twice per week for 3 consecutive weeks (*n* = 6). (a) Growth curves of SKOV3 subcutaneous xenograft tumors. (b) Representative images of the excised tumors from the experiment on day 28 after tumor cell injection. (c, d) The changes of miR-223 (c) and phosphatase and tensin homolog (PTEN) (d) in tumor sections of mice treated as indicated above were plotted on day 28 after tumor cell injection. (e) Representative photographs of PTEN, p-protein kinase B (AKT) and AKT protein expression of tumors collected from each group detected by western blotting. (f) The immunohistochemistry analyses for PTEN and Ki67 staining were carried out on SKOV3 xenograft tumor sections. Representative staining, 200×. (g) Terminal deoxynucleotidyl transferase dUTP nick end labeling (TUNEL) analysis in tumors treated as indicated above. 50×. ∗*P* < 0.05, ∗∗*P* < 0.01. Printed from Ref. [53] with permission. (C) (a) The internalization of immortalized ovarian surface epithelial 80 cell-derived exosomes (IOSE80-exo), SKOV3-exo and SKOV3-DDP-exo (PKH26-labeled, red fluorescence) by human umbilical vein endothelial cells (HUVECs) (Hoechst 33342-labeled, green fluorescence) was examined by confocal laser scanning microscopy. (b) Proliferation of HUVECs co-cultured with SKOV3-exo, or SKOV3-DDP-exo was measured by cell counting kit-8 (CCK-8), cells treated with PBS were served as the control. (c, d) Migration of HUVECs co-cultured with SKOV3-exo, or SKOV3-DDP-exo was measured by scratch assays. Cells treated with PBS were served as the control. (e, f) Invasion of HUVECs co-cultured with SKOV3-exo, or SKOV3-DDP-exo was measured by transwell assay, cells treated with PBS were served as the control. (g) Capillary-like tubes of HUVECs co-cultured with SKOV3-exo, or SKOV3-DDP-exo were measured by tube formation experiment, cells treated with PBS were served as the control. ∗*P* < 0.05, ∗∗*P* < 0.01, ∗∗∗*P* < 0.001. Printed from Ref. [54] with permission. (D) Cancer-associated fibroblasts (CAF) -derived exosomal miR-98-5p stimulates cisplatin resistance in ovarian cancer (OC) cells by targeting cyclin-dependent kinase inhibitor 1A (CDKN1A). A2780 cells transfected with oe-CDKN1A or oe-NC were co-cultured with CAF-exo and further treated with cisplatin. (a) Cell proliferation as detected by CCK-8 assay. (b) OC cell colony formation ability as detected by colony formation assay. (c) Statistical analysis for (b). (d) OC cell cycle distribution as detected by flow cytometry. (e) Statistical analysis for (d). (f) OC cell apoptosis as detected by flow cytometry. (g) Statistical analysis for (f). ∗*P* < 0.05 vs. NC-exo + oe-NC; ^&^*P* < 0.05 vs. NC-exo + oe-CDKN1A; cells without treatment of cisplatin. The experiment was repeated three times. Printed from Ref. [57] with permission. (E) MiR-296-3p in plasma extracellular vesicles (EVs) is closely associated with tumorigenesis and chemoresistance in patients with ovarian cancer. (a) miR-296-3p expression in the blood circulation of patients with ovarian cancer and healthy controls was analyzed using the dbDEMC database. (b) EVs isolated from the plasma of patients with ovarian cancer and healthy controls were observed by transmission electron microscope; white arrowhead signifies EVs (scale bar, 500 nm). (c) Nanoparticle tracking analysis of the isolated EVs. (d) Western blot analysis of EV marker protein expression, including cluster of differentiation 9 (CD9), heat shock protein 70 (HSP70), Alix, and Annexin-V. (e) The miR-296-3p expression in plasma EVs of patients with ovarian cancer and healthy controls was detected using real-time PCR. (f) MiR-296-3p expression in plasma EVs of drug-sensitive and drug-resistant patients with ovarian cancer. ∗*P* < 0.05; ∗∗*P* < 0.01. Printed from Ref. [58] with permission. FL2-A: fluorescence channel 2-area; FITC: fluorescein isothiocyanate.

Exosomes derived from CD163^+^ tumor-associated macrophages (TAMs) are absorbed by ovarian cancer cells, resulting in an increased expression of miR-221-3p. This upregulation leads to a decrease in a disintegrin and metalloproteinase with thrombospondin motifs 6 (ADAMTS6) levels and activates the protein kinase B (AKT) signaling pathway. Moreover, miR-221-3p enhances the expression of EMT transcription factors such as snail family transcriptional repressor 1 (SNAIL1) and zinc finger E-box binding homeobox 1 (ZEB1), along with the mesenchymal marker vimentin (VIM), while reducing the expression of the epithelial marker E-cadherin [52] (Table 1 and Fig. 4A). These changes induce EMT, promoting the acquisition of a tumor stem-like phenotype, and enhance the proliferation, adhesion, migration, and drug resistance of epithelial ovarian cancer (EOC) cells.

**Table 1:** Exosome miRNA and chemoresistance.

mRNA	Pathways or targets	Role	Refs.
miR-221-3p	ADAMTS6	Activate AKT	[52]
miR-223	PTEN/PI3K/AKT	Trigger resistance	[53]
miR-130a	High expression	Promotion of angiogenesis	[54]
miR-21	APAF1	Reduction of metastasis	[55]
miR-21-5p	PDHA1	Inhibits chemosensitivity	[56]
miR-98-5p	CDKN1A	Promote cisplatin resistance	[57]
miR-296-3p	PTEN/SOCS6	Promote resistance	[58]
miR-6836	DLG2/Yap1/TEAD1	Promote resistance	[59]
miR-1246	Cav1	Promote resistance	[60]
miR-146a	PI3K/Akt	Reduce resistance	[61]
miR-429	CASR/STAT3	Promote resistance	[62]
miR-675-3p	To be studied	Promote resistance	[63]
miR-181c	Wnt/β-catenin	Promote chemosensitivity	[64]

ADAMTS6: a disintegrin and metalloproteinase with thrombospondin motifs 6; AKT: protein kinase B; PTEN: phosphatase and tensin homolog; PI3K: phosphoinositide 3-kinase; APAF1: apoptotic protease activating factor 1; PDHA1: pyruvate dehydrogenase E1 alpha subunit 1; CDKN1A: cyclin-dependent kinase inhibitor 1A; SOCS6: suppressor of cytokine signaling 6; DLG2: discs large MAGUK scaffold protein 2; Yap1: yes-associated protein 1; TEAD1: TEA domain transcription factor 1; Cav1: caveolin 1. CASR: calcium-sensing receptor; STAT3: signal transducer and activator of transcription 3; Wnt: wingless/integrated.

Under hypoxic conditions, ovarian cancer cells can polarize macrophages towards an M2 phenotype. This process is accompanied by an increased expression of miR-223 in TAM-derived exosomes, which are then internalized by EOC cells, promoting a chemoresistant phenotype. The newly identified exosomal miR-223/phosphatase and tensin homolog (PTEN)/phosphoinositide 3-kinase (PI3K)/AKT signaling axis significantly contributes to drug resistance in these cells [53] (Table 1 and Fig. 4B).

Exosomes released from cisplatin (DDP)-resistant SKOV3 cells (SKOV3-DDP-exo) and DDP-sensitive SKOV3 cells (SKOV3-exo) have been studied for their effects on angiogenesis. Both types of exosomes are internalized by endothelial cells, leading to enhanced proliferation, migration, invasion, and tube formation. Notably, SKOV3-DDP-exo exhibits a more potent angiogenic effect, indicating that it contains specific components with strong pro-angiogenic activity. It has been revealed that miR-130a is highly expressed in drug-resistant ovarian cancer cells, particularly in SKOV3-DDP-exo compared to SKOV3-exo, suggesting its pivotal role in promoting angiogenesis in chemoresistant ovarian cancer cells [54] (Table 1 and Fig. 4C).

Exosomal miR-21 has been shown to modulate the oncogenic potential of metastatic ovarian cancer cells by directly regulating apoptotic protease activating factor 1 (APAF1) mRNA expression through binding to the APAF1 coding sequence. APAF1 is a direct downstream target of miR-21 [55] (Table 1). Inhibiting stroma-derived miR-21 presents a promising therapeutic approach for addressing metastatic and recurrent ovarian cancer. Moreover, exosomal miR-21-5p, originating from cisplatin-resistant SKOV3 ovarian cancer cells, enhances glycolysis and diminishes chemosensitivity in parental SKOV3 cells through the targeting of pyruvate dehydrogenase E1 alpha subunit 1 (PDHA1). This finding underscores the significance of the miR-21-5p/PDHA1 axis in ovarian cancer and suggests new avenues for therapeutic strategies [56] (Table 1).

Cancer-associated fibroblasts (CAFs) are a prominent stromal cell type in various cancers, including ovarian cancer. Exosomes derived from CAFs contain overexpressed miR-98-5p, which has been shown to enhance cisplatin resistance in ovarian cancer by downregulating cyclin-dependent kinase inhibitor 1A (CDKN1A). This downregulation reduces apoptosis, facilitating the survival of cancer cells under chemotherapeutic pressure. Targeting CAF-derived exosomal miR-98-5p may provide a novel approach for treating ovarian cancer by overcoming drug resistance [57] (Table 1 and Fig. 4D).

Additionally, activated CAFs release exosomes enriched with miR-296-3p, which facilitates ovarian cancer progression. MiR-296-3p directly targets phosphatase and tensin homolog (PTEN) and suppressor of cytokine signaling 6 (SOCS6), leading to the activation of the AKT and signal transducer and activator of transcription 3 (STAT3) signaling pathways. This activation promotes tumor cell survival and proliferation, contributing to the chemoresistant phenotype observed in ovarian cancer patients. Elevated plasma levels of miR-296-3p in EVs have been strongly correlated with both tumorigenesis and resistance to chemotherapy [58] (Table 1 and Fig. 4E).

**Fig. 5:**
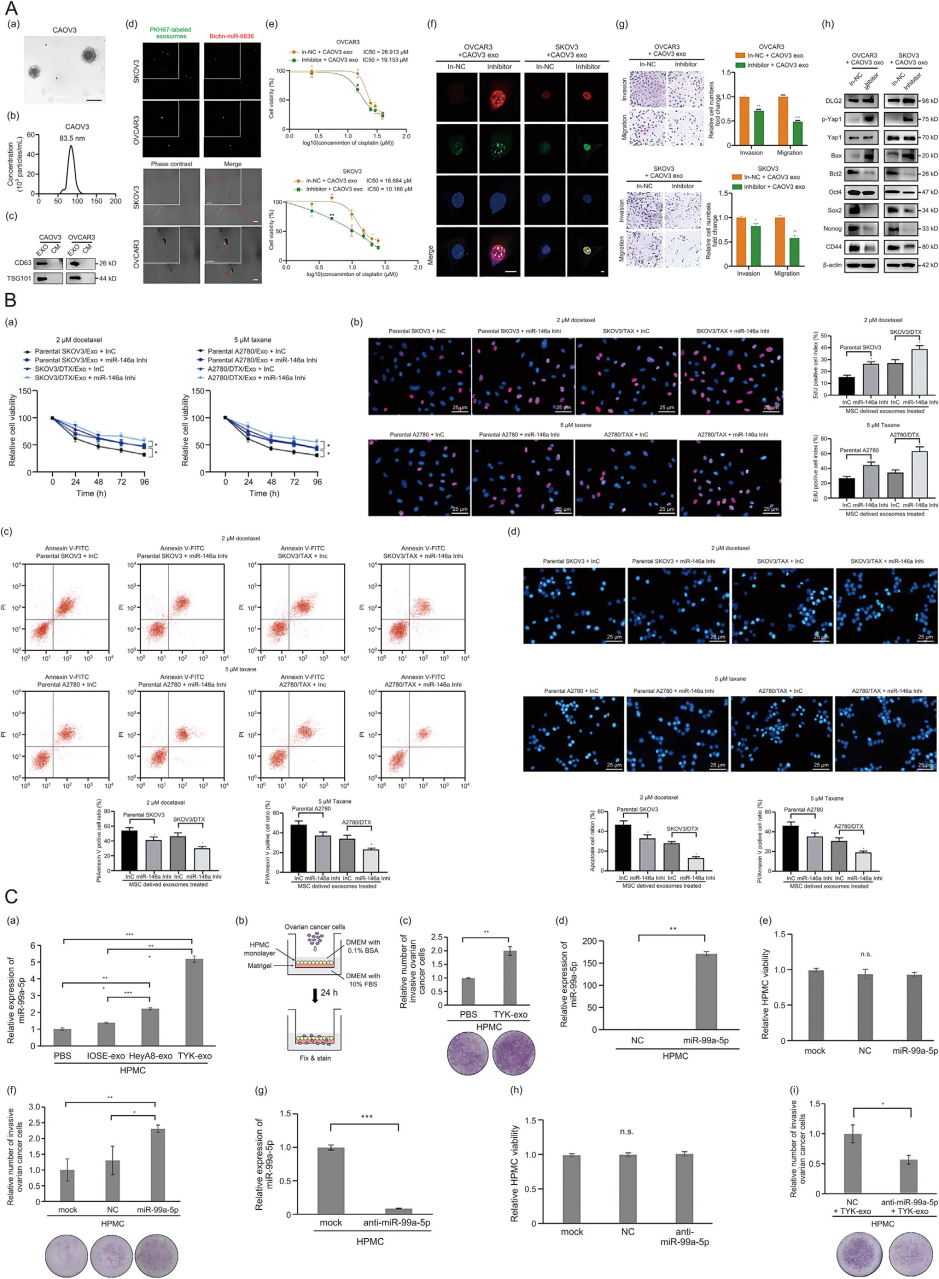
Exosomal microRNA (miRNAs) modulate cisplatin resistance in ovarian cancer. (A) Intercellular transfer of miR-6836 by exosomes disseminates cisplatin resistance. (a) Representative carcinoma ovarian 3 (CAOV3) exosome image under electron microscopy. Scale bar, 100 nm. (b) Nanoparticle tracking analysis measuring the diameter of CAOV3 exosomes. (c) Western blotting analysis of exosomal protein markers, cluster of differentiation 63 (CD63) and tumor susceptibility gene 101 (TSG101) expression in exosomes and culture medium after exosome purification gathered from CAOV3 and ovarian cancer 3 (OVCAR3) culturing. (d) Exosome internalization: CAOV3 exosomes (Green, PKH67-labeled) containing biotin-miR-6836 (Red, Streptavidin/RBITC-stained) were uptake by OVCAR3/SKOV3 (Phase contrast) cells. Scale bar, 10 μm. (e) Cell counting kit-8 (CCK-8) assay to evaluate the cell viability of OVCAR3/SKOV3 treated with inhibitor/in-negative control (NC) and CAOV3 exosomes and further treated with cisplatin at various concentrations. Half maximal inhibitory concentration (IC_50_) calculation to evaluate the effect of exosomes on transferring the cisplatin sensitivity of epithelial ovarian cancer (EOC) cells by SPSS. (f) Immunofluorescence assay for phosphorylated histone H2AX (γ-H2AX) and tumor protein p53 binding protein 1 (53BP1) expression representing DNA damage caused by cisplatin treatment for 24 h. Scale bar, 10 μm. (g) Transwell assay to analyze invasion and migration ability of OVCAR3/SKOV3 treated with inhibitor/in-NC and CAOV3 exosomes. Representative images of transwell assay are shown on the left and quantitative analyses are presented as histograms. (h) Western blot analysis of discs large MAGUK scaffold protein 2 (DLG2), p-yes-associated protein 1 (Yap1), Yap1, BCL-2 associated X protein (Bax), B-cell lymphoma 2 (Bcl2), octamer-binding transcription factor 4 (Oct4), Nanog, CD44, SRY-box transcription factor 2 (Sox2) and β-actin expression in OVCAR3/SKOV3 treated with inhibitor/in-NC and CAOV3 exosomes. Results are presented as mean ± standard error of the mean (SEM); ∗*P* < 0.05, ∗∗*P* < 0.01, ∗∗∗*P* < 0.001. Printed from Ref. [59] with permission. (B) Exosomes from human umbilical cord mesenchymal stem cells (hUCMSCs) promote OVCAR3 cell chemosensitivity partly by transferring miR-146a. hUCMSC-derived exosome-treated parental or drug resistant OC cells were exposed to 2 µM docetaxel (DTX) or 5 µM paclitaxel (TAX) for 2 h, respectively. (a) Cell viability assessed by CCK-8 cell viability assays. (b) 5-Ethynyl-2'-deoxyuridine (EdU) staining for cell proliferation. Scale bar, 25 µm. (c) Flow cytometry analysis for cells labelled with propidium iodide (PI)/Annexin V. (d) Hoechst 33258 staining for cell apoptosis. Scale bar, 25 µm. Data are presented as the mean ± standard deviation (SD). One-way (panels (c), (d) and (e)) or two-way (panel (a)) analysis of variance (ANOVA) followed by Tukey's post hoc test was used to determine statistical significance. Each assessment was performed in triplicate with three repetitions to ensure minimum deviation. ∗*P* < 0.05 vs. parental cells co-cultured with exosomes derived from InC-transfected mesenchymal stem cells (MSCs). Printed from Ref. [61] with permission. (C) Exosomal transfer of miR-99a-5p to human peritoneal mesothelial cells (HPMCs) promotes EOC cell invasion. (a) miRNA qRT-PCR. Relative expression levels of miR-99a-5p in HPMCs treated with immortalized ovarian surface epithelial cells (IOSE)- and EOC-derived exosomes at 100 μg/mL for 24 h are shown. Expression levels of miR-99a-5p in HPMCs treated with phosphate buffered saline (PBS) alone were set to 1.0. Data represent the mean ± SD of three experiments. (b) A schematic of the HPMC-coated *in vitro* invasion assay. HPMCs were plated onto 24-well cell culture inserts coated with matrigel. After HPMCs reached confluence, cancer cells were incubated on the HPMCs monolayer and allowed to invade for 24 h. Cells that migrated to the trans-side of the membrane were quantitated. (c) HPMCs were pre-cultured with TYK-nu-derived exosomes at 100 μg/mL for 24 h and then TYK-nu cells were plated onto the HPMC monolayer. Twenty-four hours after incubation, invading cells on the underside of the filter were counted. Representative pictures of the chambers are shown below. (d) miRNA qRT-PCR. Relative expression levels of miR-99a-5p in HPMCs transfected with miR-99a-5p are shown. (e) *In vitro* cell proliferation assay. HPMCs were transfected with negative control miRNA or precursor miR-99a-5p. Twenty-four hours later, relative cell proliferation was assessed using the MTS assay. (f) HPMC-coated *in vitro* invasion assay. After HPMCs were transfected with negative control miRNA or precursor miR-99a-5p, TYK-nu cells were plated onto the HPMC monolayer and allowed to invade. (g) miRNA qRT-PCR. Relative expression levels of miR-99a-5p in HPMCs transfected with anti-miR-99a-5p are shown. (h) *In vitro* cell proliferation assay. HPMCs were transfected with negative control miRNA and anti-miR-99a-5p. (i) HPMC-coated *in vitro* invasion assay. After HPMCs transfected with negative control miRNA or precursor anti-miR-99a-5p were pre-cultured with TYK-exo, TYK-nu cells were plated onto the HPMC monolayer and allowed to invade. Data represent the mean ± SD of three experiments. ∗*P* < 0.05, ∗∗*P* < 0.01, and ∗∗∗*P* < 0.001. Printed from Ref. [62] with permission. DAPI: 4',6-diamidino-2-phenylindole; FITC: fluorescein isothiocyanate; DMEM: Dulbecco's modified eagle medium; BSA: bovine serum albumin; FBS: fetal bovine serum.

High-throughput screening has identified miR-6836 as a miRNA associated with stemness and chemoresistance in drug-resistant ovarian cancer tissues. MiR-6836 directly targets discs large MAGUK scaffold protein 2 (DLG2), promoting the nuclear translocation of yes-associated protein 1 (Yap1). Yap1, regulated by TEA domain transcription factor 1 (TEAD1), establishes a positive feedback loop, reinforcing chemoresistance. Exosomal miR-6836 from cisplatin-resistant cells can be transferred to cisplatin-sensitive cells, conferring resistance to these previously sensitive cells. This transfer highlights the role of exosomal microRNA (miRNAs) in spreading resistance traits within the tumor microenvironment [59] (Table 1 and Fig. 5A).

MiR-1246 has been identified as a highly expressed miRNA in exosomes derived from paclitaxel-resistant ovarian cancer cells. It directly targets the *Cav1* gene, which plays a role in the transfer of exosomes and cell signaling. The elevated expression of miR-1246 in exosomes from resistant cells suggests its involvement in promoting resistance to paclitaxel. Treatment strategies enhancing Cav1 levels or targeting miR-1246 could potentially increase the sensitivity of ovarian cancer cells to paclitaxel, providing a promising therapeutic direction [60] (Table 1).

Exosomal miR-146a, derived from human umbilical cord mesenchymal stem cells (hUCMSCs), has emerged as a potential modulator of chemoresistance in ovarian cancer. The introduction of exosomes containing miR-146a into drug-resistant cells results in a marked decrease in cell proliferation and enhanced sensitivity to chemotherapy. This effect is mediated through the downregulation of laminin subunit γ 2 (LAMC2), a component of the extracellular matrix, which is involved in cell adhesion and signaling. The suppression of the PI3K/AKT signaling pathway by miR-146a further sensitizes cancer cells to chemotherapeutic agents, demonstrating the therapeutic potential of targeting exosomal miRNAs to overcome drug resistance [61] (Table 1).

Microarray analyses have revealed that miR-429 is upregulated in multidrug-resistant SKOV3 cells and their exosomes compared to sensitive A2780 cells and their exosomes. Exosomal miR-429 has been shown to promote proliferation and drug resistance in A2780 cells by targeting the calcium-sensing receptor (CASR) and activating the STAT3 pathway, both *in vitro* and *in vivo*. This miRNA’s role in regulating the CASR/STAT3 axis indicates its potential as a therapeutic target to counteract chemoresistance in ovarian cancer [62] (Table 1 and Fig. 5B).

Furthermore, exosomes derived from cisplatin-sensitive (A2780) and cisplatin-resistant (A2780/DDP) cells, designated as Exo-A2780 and Exo-A2780/DDP, respectively, have been studied to understand the role of exosomal miRNAs in chemoresistance. Analysis of these exosomes revealed that hsa-miR-675-3p is associated with drug resistance. This miRNA may serve as a promising therapeutic target for overcoming cisplatin resistance in ovarian cancer. Further research is needed to elucidate the specific mechanisms by which hsa-miR-675-3p contributes to chemoresistance, but its presence in exosomes highlights the importance of miRNA-mediated intercellular communication in cancer therapy [63] (Table 1).

Exosomes carrying miR-181c from bone marrow stromal cells (BMSCs) have been shown to influence cisplatin resistance in ovarian cancer cells. Overexpression of miR-181c downregulates mesoderm specific transcript (MEST) expression and reduces resistance by inhibiting the wingless/integrated (Wnt)/β-catenin signaling pathway. The delivery of miR-181c through EVs derived from BMSCs highlights the potential of exosomal miRNAs in reversing drug resistance and providing new therapeutic avenues for treating ovarian cancer [64] (Table 1).

### 5.2 circ-RNA in exosomes and chemoresistance

**Fig. 6:**
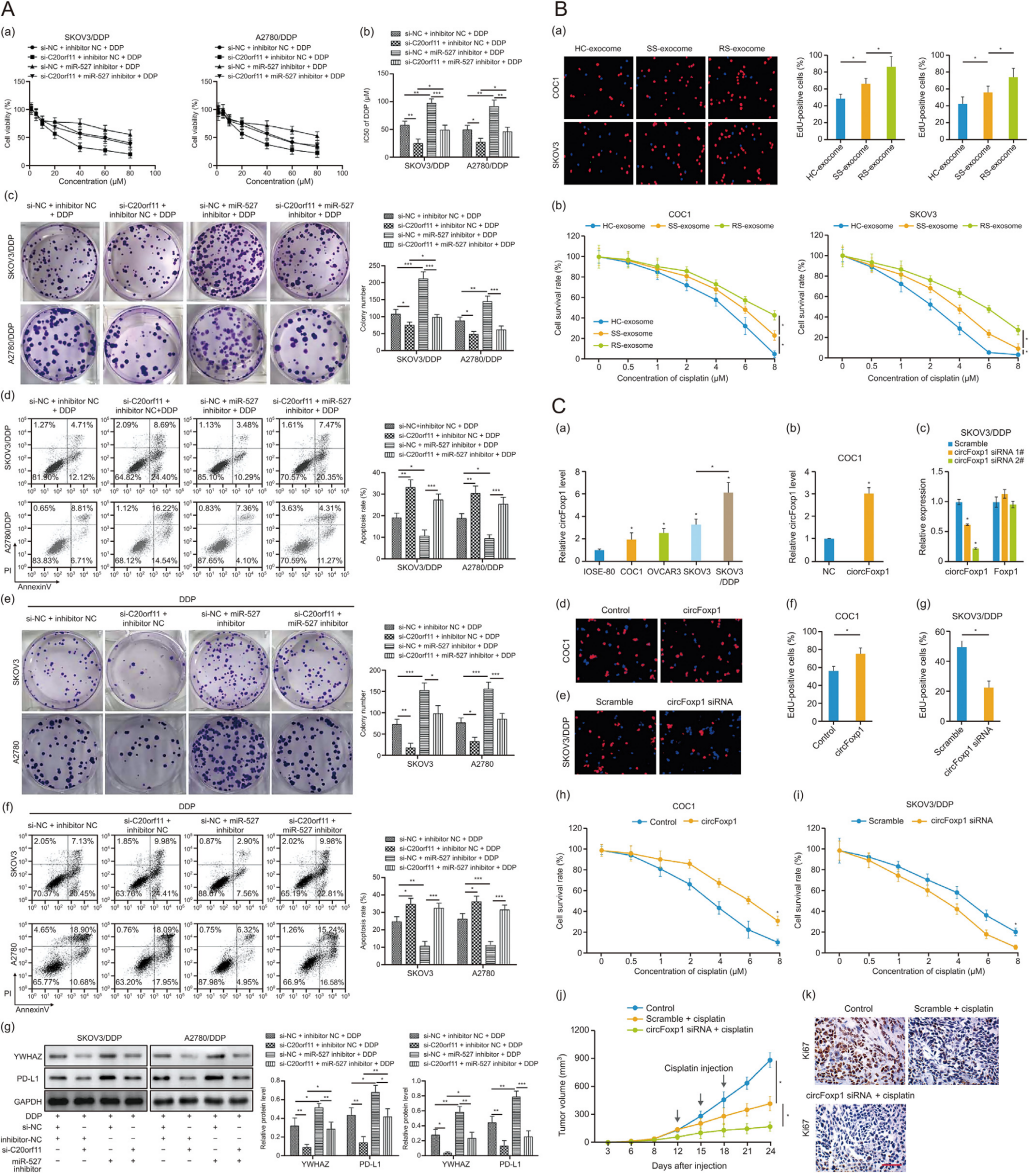
Circular RNAs in exosomes mediate cisplatin resistance in ovarian cancer. (A) circ_C20orf11 promotes cisplatin (DDP) resistance in SKOV3/DDP and A2780/DDP cells by sponging miR-527. (a) After SKOV3/DDP and A2780/DDP cells were transfected with adenoviral circ_C20orf11 or its vector control and miR-527 inhibitor, the cells were treated with DDP. The viability of SKOV3/DDP or A2780/DDP cells with or without adenoviral C20orf11 (si-C20orf11 + inhibitor + negative control (NC) + DDP) or its vector control (si-NC + inhibitor + NC + DDP) and miR-527 inhibitor transfection. (b) half maximal inhibitory concentration (IC_50_) of DDP using the same conditions as described in (a). (c) Cisplatin colony formation assays were performed using the same conditions as described in (a). (d) Cell apoptosis assays were performed using the same conditions as described in (a). (e) The relative colony number ratio in DDP-treated SKOV3 and A2780 cells with adenoviral C20orf11/miR-527 inhibitor (si-C20orf1 + DDP + miR-527 + DDP) or with adenoviral C20orf11/miR-527 inhibitor negative control (si-C20orf11 + inhibitor NC + DDP) or vector control transfection/miR-527 inhibitor (si-NC + miR-527 inhibitor + DDP) and the vector control transfection/miR-527 inhibitor negative control (si-NC+inhibitor NC+ DDP). (f) The apoptosis rate in DDP-treated SKOV3 and A2780 cells treated with adenoviral C20orf11/miR-527 inhibitor (si-C20orf1 + DDP + miR-527 + DDP) or with adenoviral C20orf11/miR-527 inhibitor negative control (si-C20orf11 + inhibitor NC + DDP) or vector control transfection/miR-527 inhibitor (si-NC+miR-527 inhibitor+ DDP) and the vector control transfection/miR-527 inhibitor negative control (si-NC + inhibitor NC + DDP). (g) Western blot analyses using the same conditions as described in (a). *n* = 3. ∗*P* < 0.05, ∗∗*P* <0.01, ∗∗∗*P* < 0.001. Printed from Ref. [65] with permission. (B) The effects of exosomes on cell survival and proliferation. (a) Representative images of 5-Ethynyl-2'-deoxyuridine (EdU) staining, and quantification of the EdU-positive cells (right). (b) The survival rate in cells treated with exosomes (1 × 107 particles/mL) and cisplatin. Printed from Ref. [68] with permission. (C) circFoxp1 knockdown attenuates cisplatin-resistance in epithelial ovarian cancer (EOC) cells. (a) The expression of circFoxp1 in EOC cell lines. (b) The expression of circFoxp1 in COC1 cells after transfection. (c) The expression of circFoxp1 and Foxp1 in SKOV3/DDP cells after siRNA transfection. (d, e) Representative images of EdU staining in COC1 cells (d) and SKOV3/DDP cells (e). (f, g) quantification of the EdU-positive cells in (d) and (e). (h, i) The survival rate in COC1 cells (h) and SKOV3/DDP cells (i). (j) The tumor volumes of each group. (k) The expression of Ki67 in xenografted tumor tissues. Scale bar, 200 μm. Printed from Ref. [68] with permission. circFoxp1: circular forkhead box protein P1; GAPDH: glyceraldehyde-3-phosphate dehydrogenase; PD-L1: programmed death-ligand 1; IOSE-80: immortalized ovarian surface epithelial cells-80.

Recent studies have demonstrated the significant role of circRNAs encapsulated within exosomes in modulating chemoresistance in ovarian cancer. One notable circRNA, circ_C20orf11, has been shown to contribute to cisplatin resistance by inhibiting miR-527 and upregulating tyrosine 3-monooxygenase/tryptophan 5-monooxygenase activation protein zeta (YWHAZ) expression. This pathway promotes the polarization of macrophages towards an M2 phenotype, which is associated with a tumor-promoting environment. The presence of circ_C20orf11 in exosomes suggests its potential as a biomarker for predicting drug resistance in ovarian cancer patients [65] (Fig. 6A).

Another circRNA, circ_0025033, exhibits high expression in paclitaxel-resistant ovarian cancer cells and tissues. Mechanistic studies have revealed that circ_0025033 acts by sponging miR-532-3p, thereby upregulating forkhead box protein M1 (FOXM1) expression. This interaction leads to enhanced resistance to paclitaxel. Knockdown of circ_0025033 reduces paclitaxel resistance by decreasing FOXM1 levels and increasing miR-532-3p, highlighting its role in the malignant behavior of ovarian cancer cells under chemotherapy [66].

Elevated levels of hsa_circ_0010467 in serum exosomes of patients with platinum-resistant ovarian cancer are correlated with advanced tumor stages and poor prognosis. This circRNA promotes resistance to platinum-based chemotherapy through the AU-rich element RNA-binding protein 1 (AUF1)/hsa_circ_0010467/miR-637/leukemia inhibitory factor (LIF)/STAT3 axis. The study suggests that targeting hsa_circ_0010467 could serve as a therapeutic strategy for overcoming platinum resistance in ovarian cancer patients [67].

Exosomal circFoxp1 is markedly upregulated in EOC patients resistant to cisplatin treatment. This circRNA enhances cell proliferation and cisplatin resistance, possibly by regulating the expression of CCAAT/enhancer-binding protein γ (CEBPG) and formin-like protein 3 (FMNL3) through miR-22 and miR-150-3p. The oncogenic role of circFoxp1 in EOC cells makes it a potential biomarker and therapeutic target for managing resistance in these patients [68] (Figs. 6B and C).

Lastly, circ-PIP5K1A, identified as a contributor to ovarian cancer tumorigenesis, has been implicated in promoting cisplatin resistance. This circRNA operates by sequestering miR-942-5p, thus upregulating nuclear factor I B (NFIB) expression. The transfer of circ-PIP5K1A via exosomes from resistant to sensitive cells highlights the mechanism of exosomal circRNAs in spreading chemoresistance, presenting novel targets for therapeutic intervention in ovarian cancer [69].

### 5.3 Proteins in exosomes and chemoresistance

**Fig. 7:**
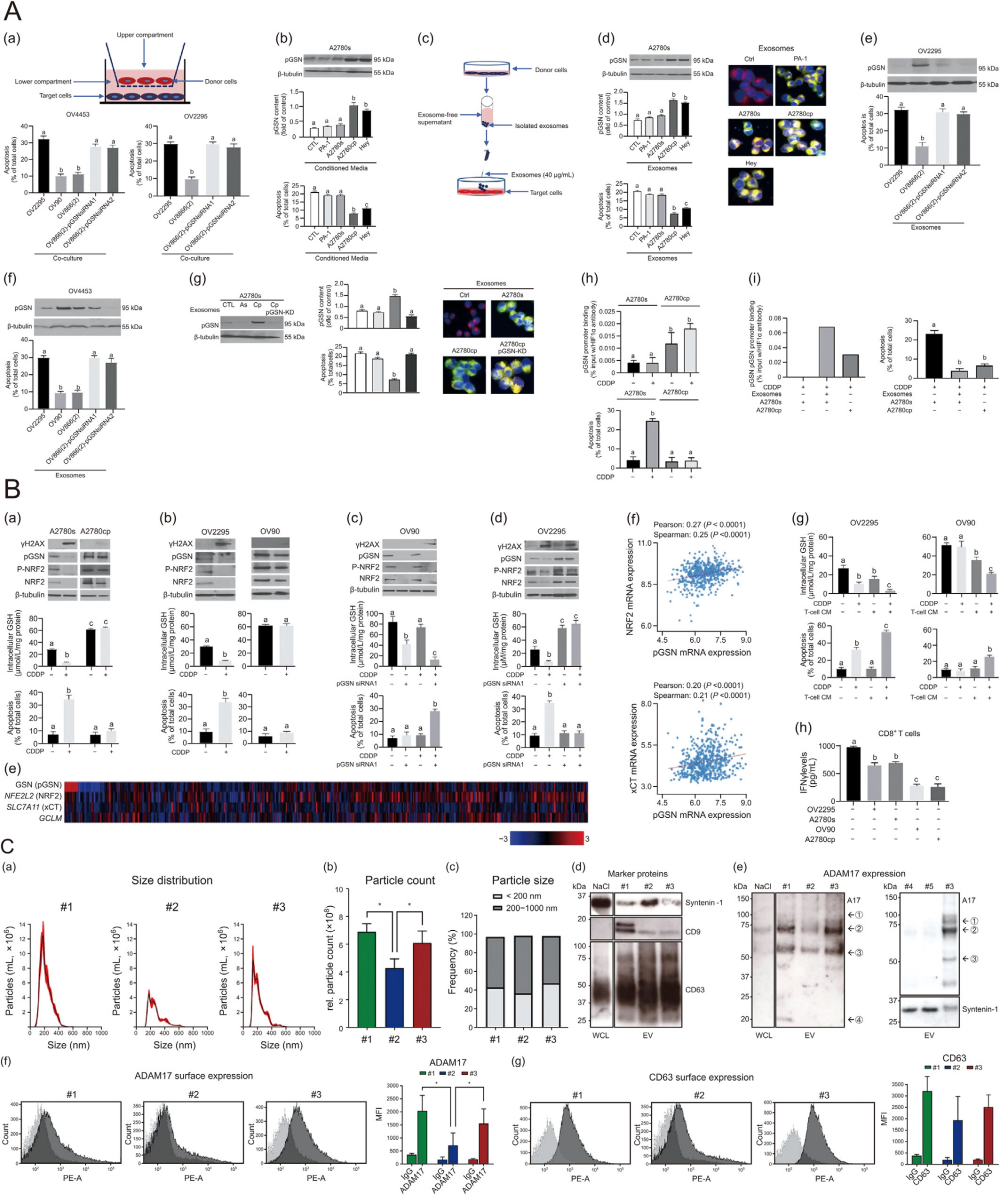
Exosomes promote chemoresistance via molecular pathways in ovarian cancer. (A) Chemoresistant cells-derived exosomes enhance hypoxia-inducible factor 1α (HIF1α) binding to plasma gelsolin (pGSN) promoter region and induces cisplatin (DDP) resistance in chemosensitive ovarian cancer (OVCA) cells. (a) Chemosensitive OVCA cells (target cells; OV4453, OV2295) were co-cultured with chemoresistant OVCA cells (OV90, OV866(2)), chemosensitive OVCA cells (OV2295), and pGSN-knocked down OV866(2) cells followed by DDP treatment (10 µm; 24 h). Chemoresistant (OV90 and OV866(2)) but not the chemosensitive OVCA cells conferred DDP resistance to chemosensitive OVCA cells. OV866(2) cells whose pGSN was knocked down failed to protect OV4453 and OV2295 against DDP-induced apoptosis. (b, c) Conditioned media and exosomes from chemoresistant OVCA cells but not the chemosensitive cells increased pGSN content and conferred DDP resistance to chemosensitive cells. A2780s cells were treated with conditioned media (b, 3 mL; 24 h) or exosomes (c–g, 40 µg/400,000 cells; 24 h) derived from cultures of A2780s, PA-1, Hey, OV2295, OV866(2), OV90, and A2780cp cells, and then cultured with or without DDP (10 µM; 24 h). Exosomes were tagged with pCT-CD63-GFP (1 µg; 24 h) and their uptake by recipient cells (A2780s, labeled with PKH26 red fluorescent dyes) was assessed by IF. (e, f) Exosomes from chemoresistant cells depleted of pGSN failed to upregulate pGSN content and facilitated DDP-induced apoptosis compared with exosomes with pGSN. Exosomal pGSN from chemoresistant OVCA cells confer resistance in OV2295 and OV4453 cells. (g) A2780s cells were cultured with exosomes (40 µg/400,000 cells; 24 h) derived from A2780s, A2780cp, and A2780cp following pGSN knockdown (A2780cp-pGSN-KD) after which they were treated with or without DDP (10 µM; 24 h). pGSN and β-tubulin contents (loading control) were examined by WB. (h) HIF1α-pGSN promoter binding is higher in A2780cp than A2780s cells. A2780s and A2780cp cells were cultured with or without DDP (10 µM; 24 h) and HIF1α-pGSN promoter binding was assessed by the CHIP assay. (i) Chemoresistant cells-derived exosomes increase HIF1α-pGSN promoter binding and attenuate DDP-induced apoptosis in chemosensitive cells. A2780s cells were cultured with A2780cp cells-derived exosomes (40 µg/400,000 cells; 24 h), and then cultured with or without DDP (10 µM; 24 h). HIF1α-pGSN promoter binding was assessed by chip assay. ((a) a; ∗∗∗*P* < 0.001 vs. b; (b) a; ∗∗∗*P* < 0.001 vs. b and c; (d) a; ∗∗∗*P* < 0.001 vs. b and c; (e) a; ∗∗∗*P* < 0.001 vs. b; (f) a; ∗∗∗*P* < 0.001 vs. b; (g) a; ∗∗∗*P* < 0.001 vs. b); (h) a; ∗∗*P* < 0.01 vs. b); (i) a; ∗∗*P* < 0.01 vs. b). *n* = 3. Printed from Ref. [70] with permission. (B) Increased pGSN expression promotes nuclear factor erythroid 2-related factor 2 (NRF2)-dependent glutathione (GSH) production. (a) Chemosensitive (A2780s) and chemoresistant (A2780cp) paired cell lines were treated with or without DDP (10 μmol/L, 24 h). (b) Chemosensitive (OV2295) and chemoresistant (OV90) high-grade serous (HGS) ovarian cancer cell lines were treated with or without DDP (10 μmol/L, 24 h). (c) pGSN expression in chemoresistant HGSC cell line (OV90) was silenced (siRNA1; 50 nmol/L, 24 h) and treated with or without DDP (10 μmol/L, 24 h). (d) pGSN was force expressed in chemosensitive HGSC cell line (OV2295; cDNA, 2 μg, 24 h) and treated with or without DDP (10 μmol/L, 24 h). (e, f) Heatmap (e) and Pearson/Spearman analysis (f) of the association between NFR2-dependent mRNAs (NRF2 and cystine/glutamate transporter (xCT)) and pGSN mRNA expression. (g) Chemosensitive (OV2295) and chemoresistant (OV90) cell lines were primed with CM (3 mL, 24 h) from activated CD8^+^ T cells and then treated with or without DDP (10 μmol/L, 24 h). (h) Activated human peripheral CD8^+^ T cells were cocultured with chemosensitive (OV2295 and A2780s) and chemoresistant (OV90 and A2780cp) cell lines (24 h). mRNA analysis was interrogated using The Cancer Genome Atlas (TCGA) datasets on cBioPortal. Intracellular GSH was measured by colorimetric assays and Western blotting was used to examine protein contents (phosphorylated histone H2AX (γH2AX), pGSN, pNRF2, NRF2, and β-tubulin). Apoptosis was measured morphologically by Hoechst staining and interferon γ (IFNγ) levels were assayed using sandwich enzyme-linked immunosorbent assay (ELISA). Results are expressed as means ± standard deviation (SD) from three independent replicate experiments. (a) a, ∗∗∗*P* < 0.001 vs. b and c; (b) a, ∗∗∗*P* < 0.001 vs. b; (c) a, ∗∗*P* < 0.01 vs. b and a, ∗∗∗*P* < 0.001 vs. c; (d) a, ∗∗∗*P* < 0.001 vs. b and c; (g) a, ∗∗*P* < 0.01 vs. b and a, ∗∗∗*P* < 0.001 vs. c; (h) a, ∗∗*P* < 0.01 vs. b and a, ∗∗∗*P* < 0.001 vs. c. Printed from Ref. [71] with permission. (C) Native ascites-derived extracellular vesicles (EVs) contain mature a disintegrin and metalloprotease 17 (ADAM17). Ascites-EV derived from three ovarian cancer patients (#1, #2, #3) were isolated by differential ultracentrifugation and characterized. Size distribution and concentration of ascites-EV were determined by nanoparticle tracking analysis (NTA) and displayed as (a) histograms of size distribution, (b) bar charts of concentration and (c) the relative fraction of EV < 200 nm and 200–1,000 nm in size. Size distribution and EV concentration were adjusted to the EV extracted from 1 mL ascites. (d) Marker protein expression of Syntenin-1, cluster of differentiation 9 (CD9) and CD63 was analysed in EV and whole cell lysates (WCL) by Western blotting assay. (e) Relative ADAM17 abundance in purified EV was assessed by WB analysis. EV purified from 0.5 mL ascites were loaded on the gel. Pro-form of ADAM17 (1), mature form (2) and fragments at approx. 65 kDa (3) and approx. 20 kDa (4) were detected. (f) As benign controls, flushing fluid derived from patients undergoing surgical procedures for benign gynaecological diseases such as fibroids were used (#4 and #5). The maximum amount of EV derived from 1.4 to 1.9 mL flushing fluid was loaded on the gel. Marker protein and ADAM17 (A17) expression were analyzed in comparison to EV derived from patient #3. The marker protein Syntenin-1 and ADAM17 (A17) are displayed and run in the same blot as EV derived from patient #3 for a direct comparison. (g) Surface expression of ADAM17 and CD63 on ascites-EV was evaluated via flow cytometry (FC) using phycoerythrin (PE)-conjugated anti-ADAM17 and anti-CD63 antibodies. FC data are presented as mean fluorescence intensities (MFI). NTA and FC data are represented as mean + standard error of the mean (SEM) of three independent experiments. Statistical significance is displayed as ∗*P* < 0.05. Printed from Ref. [72] with permission. SLC7A11: solute carrier family 7 member 11; GCLM: glutamate-cysteine ligase modifier subunit; PE-A: phycoerythrin-area.

Plasma gelsolin (pGSN), an isoform of the gelsolin (GSN) gene that functions as a multifunctional actin-binding protein, is secreted and transferred by exosomes. This process is implicated in the development of chemotherapy resistance in ovarian cancer. Cytosolic GSN modulates the behavior of gynecological tumor cells, resulting in altered sensitivity to chemotherapy. Specifically, exosome-mediated secretion and transport of pGSN lead to the upregulation of hypoxia-inducible factor 1-α (HIF1α)-mediated pGSN expression in chemoresistant ovarian cancer cells in an autocrine manner. This upregulation contributes to cisplatin resistance in chemosensitive ovarian cancer cells. Targeting pGSN may offer a promising approach for personalized treatment of chemotherapy resistance, potentially reversing the sensitivity of resistant cells [70] (Fig. 7A).

Furthermore, research has highlighted the immunomodulatory function of small extracellular vesicle plasma gelsolin (sEV-pGSN) in chemoresistance development in ovarian cancer. In chemosensitive environments, sEV-pGSN is released at reduced rates, enhancing CD8^+^ T cell functionality. This increase leads to elevated secretion of interferon gamma (IFNγ) by T cells, subsequently decreasing intracellular glutathione (GSH) production and making the cells more susceptible to cisplatin-induced apoptosis. Conversely, in chemoresistant settings, heightened secretion of sEV-pGSN by ovarian cancer cells induces apoptosis in CD8^+^ T cells, diminishing IFNγ secretion, and increasing GSH production. This process ultimately confers resistance to cisplatin-induced cell death in ovarian cancer cells. The modulation of immune surveillance and GSH biosynthesis by sEV-pGSN plays a significant role in chemotherapy resistance in ovarian cancer [71] (Fig. 7B).

A disintegrin and metalloprotease 17 (ADAM17), another protein associated with chemoresistance, enhances tumor cell proliferation and survival via the epidermal growth factor receptor (EGFR) pathway by cleaving various ligands such as amphiregulin (AREG). Research has demonstrated that enzymatically active ADAM17 is secreted in EVs from ovarian cancer cells following chemotherapy. EVs containing ADAM17 from patient-derived samples have been shown to promote AREG shedding and restore chemotherapy resistance in cells lacking ADAM17. This indicates that EVs containing ADAM17 can transmit chemotherapy resistance in ovarian cancer. Consequently, ADAM17 levels may serve as a biomarker for monitoring tumor progression and assessing chemotherapy sensitivity in patients [72] (Fig. 7C).

The role of DNA methyltransferase 1 (DNMT1) in chemotherapy resistance is also significant. Increased expression of DNMT1 has been observed in ovarian cancer tumors and their corresponding cell lines, with DNMT1 transcripts being particularly abundant in exosomes isolated from conditioned medium of ovarian cells. Exposure to these exosomes elevates endogenous DNMT1 expression and confers resistance to cisplatin-induced cytotoxicity in recipient cells. *In vivo* administration of DNMT1-containing exosomes has been shown to exacerbate xenograft progression and significantly reduce overall survival rates. Moreover, the use of the exosome inhibitor GW4869 effectively restores the sensitivity of resistant cells, suggesting that exosome inhibitors in combination with cisplatin could be a viable treatment strategy for chemotherapy-resistant ovarian cancer patients [73].

### 5.4 Exosome transport and chemoresistance

TMEM205, a recently discovered transmembrane protein, has been identified as a key factor in platinum resistance in EOC. Through its regulation of Rab11 expression, TMEM205 facilitates the development of platinum-resistant ovarian cancer by promoting exosome secretion. Recent research has focused on developing nitroxide tethering diarylpiperidone compounds that target TMEM205, demonstrating their ability to reduce exosome secretion and inhibit platinum efflux in platinum-resistant ovarian cancer. This indicates a potential therapeutic strategy to combat resistance by targeting TMEM205 [74].

*In vitro* cell culture and *in vivo* mouse model studies have shown that inhibiting O-linked β-N-acetylglucosaminylation (O-GlcNAcylation), a crucial post-translational protein modification, enhances exosome release. This increased exosome-mediated efflux of cisplatin from cancer cells contributes to the development of chemotherapy resistance. Specifically, the reduction of O-GlcNAcylation transferase (OGT) decreases O-GlcNAcylation of synaptosome-associated protein 23 (SNAP-23). This reduction facilitates the formation of a soluble SNARE complex comprising SNAP-23, vesicle-associated membrane protein 8 (VAMP8), and syntaxin 4 (Stx4) proteins, which promotes exosome release and the intracellular efflux of cisplatin, thereby contributing to chemotherapy resistance [75].

### 5.5 Hypoxic microenvironment and chemoresistance

In a hypoxic microenvironment, tumor cells exhibit increased secretion of exosomes that possess angiogenic and metastatic properties, facilitating alterations in the tumor microenvironment and promoting tumor progression [76]. The p53 pathway plays a significant role in upregulating exosome production and release during stress-inducing conditions like hypoxia and toxicity. This regulation is achieved through the transcriptional activation of various genes, including those involved in the p53-regulated tumor suppressor pathway [77]. Under hypoxic conditions, EVs from tumors can stimulate metabolic changes in glycolytic pathway proteins, enhancing resistance to carboplatin and supporting tumor advancement [78].

Exosomes derived from ascites ovarian cancer cell lines cultured in hypoxic conditions have shown enhanced expression of oncogenic proteins such as STAT3 and Fas cell surface death receptor (FAS), which contribute to increased cellular chemoresistance *in vitro*. Additionally, the efflux of cisplatin via exosomes is markedly elevated in ovarian cancer cells under hypoxic conditions. The co-culture of hypoxic exosomes (HEx) with tumor cells results in a significant reduction in double-stranded DNA damage and enhanced cell survival following cisplatin treatment. Inhibiting exosome release using established inhibitors like amiloride or targeting STAT3, in combination with cisplatin treatment, has been shown to significantly increase apoptosis and reduce clone formation and proliferation. These findings suggest that HEx may play a crucial role in enhancing chemotherapy resistance in ovarian cancer, potentially serving as a novel mechanism for tumor metastasis and resistance [79].

Hypoxia induces changes in the composition and biological functions of exosomes, leading to the development of carboplatin resistance in recipient target cells. The reduction in oxygen levels may promote tumor metastasis by stimulating the secretion of factors that support tumor growth. Research by Campos and colleagues [78]demonstrated that exosomes produced in a low-oxygen environment can trigger metabolic shifts in ovarian cancer cells, resulting in alterations in glycolytic pathway proteins that enhance carboplatin resistance. These results provide new insights into the role of EV signaling in cancer cells and highlight potential therapeutic targets for predicting and preventing disease recurrence.

### 5.6 Others

Promoter of CDKN1A antisense DNA damage-activated RNA (PANDAR) has been identified as a p53-dependent oncogene that enhances cisplatin resistance in ovarian cancer. Upon administration of cisplatin, PANDAR is transferred via exosomes, which aids ovarian cancer cells in rapidly adapting to cisplatin-induced stress through the accumulation of the serine and arginine-rich splicing factor 9 (SRSF9). These exosomes, induced by PANDAR during chemotherapy, are crucial in modulating the sensitivity of p53-mutant ovarian cancer cells to cisplatin by regulating apoptosis. Elevated levels of SRSF9 are associated with a negative prognosis in ovarian cancer, as SRSF9 promotes an increased transcriptional ratio of sirtuin 4 (SIRT4)/SIRT6. Further research into the role of SRSF9 in immune function may provide valuable insights into how this splicing factor contributes to the development of resistance to anticancer therapies within the tumor microenvironment [80].

Additionally, a specific resistant tumor cell phenotype (EpCAM^+^ CD45^+^) has been detected in the ascites of individuals diagnosed with EOC. These cells exhibit high aggressiveness and express mesenchymal genes, including subsets of ovarian cancer stem cells (CD133 ^+^ and CD117^+^CD44^+^). It has been demonstrated that EpCAM^+^CD45^+^ tumor cells have heightened expression of MHC Class I antigens, allowing them to evade immune surveillance by natural killer cells. Studies conducted on ovarian cancer-5 (OVCAR-5) cells by Ramakrishnan M. et al. [81] indicate that exosomes released by non-tumor cells present in ascites fluid play a significant role in promoting resistance and aggressiveness in cancer cells. This highlights the importance of the tumor microenvironment and the role of exosomes in facilitating the spread of resistance mechanisms.

## 6 The potential role of exosomes as carriers

Exosomes, a novel class of natural EVs, possess a bilayer membrane structure and offer unique advantages, such as high tolerance, extended circulation half-life, and efficient uptake by recipient cells [82]. These vesicles are capable of traversing physiological barriers, a property that presents significant opportunities and challenges in the development of drug delivery systems [83,84]. Additionally, exosomes can be engineered to enhance their targeting specificity, making them a versatile tool in targeted therapy. One of the key benefits of exosomes is their minimal long-term accumulation in tissues or organs, which reduces the risk of systemic toxicity compared to synthetic carriers [85].

Unlike conventional synthetic carriers such as liposomes, micelles, dendrimer polymers, and nanoparticles, exosomes are endogenous vesicles that carry a wide range of proteins and lipids. This endogenous nature contributes to their distinct mechanisms of cellular uptake and drug delivery. Notably, exosomes demonstrate superior biocompatibility and targeting abilities. Research has shown that exosomes can utilize clathrin-independent endocytosis to deliver drugs, such as cisplatin, to cisplatin-resistant cancer cells. This delivery mechanism helps evade sequestration within exosomes post-entry into the cells, ensuring a uniform distribution of the drug within cancer cells and thereby enhancing its anticancer efficacy [86].

Exosomes interact with target cells through various mechanisms [87]. These include binding to cell surfaces via specific adhesion molecule ligand-receptor pairs, direct fusion with the cell membrane, and internalization into endocytic compartments through receptor-mediated endocytosis pathways. These pathways may include caveolin- or clathrin-dependent mechanisms, lipid raft-based processes, or other methods such as phagocytosis or micropinocytosis [[88], [89], [90]]. Through these interactions, exosomes can directly stimulate target cells, facilitate the transfer of membrane receptors, and deliver molecular information carried within them to recipient cells. This capability underscores the potential of exosomes as a powerful tool in therapeutic delivery and cellular communication.

### 6.1 Exosomes as delivery tools

Numerous studies have demonstrated the potential of exosomes as nanocarriers for delivering anticancer agents such as paclitaxel (PTX) and doxorubicin (DOX), both *in vitro* and *in vivo* [91]. Exosomes have shown efficacy in transporting these drugs to multidrug-resistant cancer cells, including those that overexpress P-glycoprotein (Pgp), a protein known to contribute to drug resistance [92]. This ability to bypass conventional drug resistance mechanisms makes exosomes particularly promising for cancer therapy, allowing chemotherapeutic agents to reach their targets effectively and exert their full therapeutic effects [93,94]. Zhang et al. [95] demonstrated that exosomes derived from M1 macrophages, when loaded with cisplatin, increased the cytotoxicity against resistant A2780/DDP ovarian cancer cells by 3.3-fold and against sensitive A2780 cells by 1.4-fold, compared to treatment with cisplatin alone. This suggests that M1 exosomes can effectively enhance the delivery and efficacy of cisplatin in both resistant and sensitive ovarian cancer cell lines. Moreover, the study indicated that the cytotoxicity of cisplatin-loaded M2 exosomes was similarly increased, with 1.7-fold enhancement in A2780/DDP cells and 1.4-fold enhancement in A2780 cells [95]. These findings underscore the potential of cisplatin-loaded exosomes, particularly those derived from immune cells, as effective vehicles for chemotherapeutic delivery. Additionally, exosomal components from Anthos, a berry extract rich in anthocyanins, have been shown to increase the sensitivity of drug-resistant tumors and enhance the therapeutic efficacy of chemotherapeutic agents in the treatment of ovarian cancer [96]. This suggests that the natural bioactive compounds within exosomes can synergize with conventional drugs, providing a dual approach to overcoming resistance and improving treatment outcomes. The versatility and biocompatibility of exosomes make them an attractive option for developing advanced drug delivery systems. By leveraging their natural ability to encapsulate and protect therapeutic agents, exosomes can be engineered to deliver a wide range of drugs more efficiently and with greater specificity, potentially transforming the landscape of cancer therapy.

Several studies have shown that lncRNA associated with platinum sensitivity in ascitic exosomes (PLADE) can improve the sensitivity of high-grade serous ovarian cancer (HGSOC) to platinum-based chemotherapy. In animal models, PLADE was observed to enhance cisplatin sensitivity by facilitating the transfer of exosomes to recipient cells in culture and neighboring tumor tissues. Mechanistically, PLADE interacts with and decreases the levels of heterogeneous nuclear ribonucleoprotein D (HNRNPD) via von Hippel-Lindau protein (VHL)-mediated ubiquitination. This reduction in HNRNPD leads to an increase in RNA hybrids (R-loops) and DNA damage, thereby enhancing the sensitivity of HGSOC to cisplatin treatment [97].

**Fig. 8:**
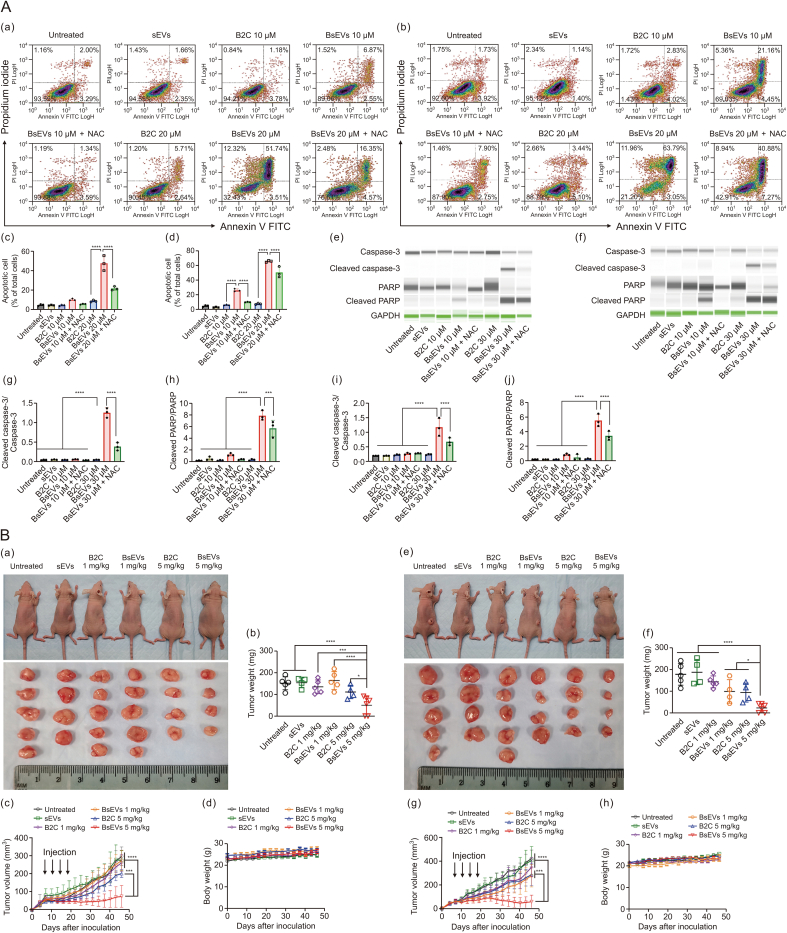
Bystander extracellular vesicles (BsEVs) induce apoptosis and suppress tumors. (A) BsEVs-induced apoptosis. (a, b) Flow cytometric analysis of OVCAR-8 cells (a) and OVCAR-8/MDR cells (b) stained with Annexin V-fluorescein isothiocyanate (Annexin V-FITC) and propidium iodide. (c, d) Quantification of BsEVs-induced apoptosis in OVCAR-8 (c) and OVCAR-8/MDR cells (d). Values are mean ± standard deviation (SD) (*n* = 3. ∗∗∗∗*P* < 0.001). (e, f) Apoptosis-related protein expression of OVCAR-8 cells (e) and OVCAR-8/MDR cells (f). (g, h) The ratio of cleaved cysteine-aspartic acid protease 3 (caspase-3)/caspase-3 (g) and cleaved poly(ADP-ribose) polymerase (PARP)/PARP (h) of OVCAR-8 cells. (i, j) The ratio of cleaved caspase-3/caspase-3 (i) and cleaved PARP/PARP (j) of OVCAR-8/MDR cells after various treatments. Values are mean ± SD (*n* = 3. ∗∗∗*P* < 0.01, and ∗∗∗∗*P* < 0.001). Printed from Ref. [98] with permission. (B) *In vivo* therapeutic anticancer activity of BsEVs. (a) Representative images of tumor-bearing mice after treatment with various doses of BsEVs in OVCAR-8. (b) Tumor weight measurement, (c) changes in tumor volume, and (d) body weights in OVCAR-8 xenograft mice. (e) Representative images of tumor-bearing mice after treatment with various doses of BsEVs in OVCAR-8/MDR. (f) Determination of tumor weight, (g) changes in tumor volume, and (h) body weights in OVCAR-8/MDR xenograft mice. Values are mean ± SD (*n* = 4–6. ∗*P* < 0.1, ∗∗∗*P* < 0.01, and ∗∗∗∗*P* < 0.001). Printed from Ref. [98] with permission. NAC: N-acetylcysteine; B2C: Benzoyl-dibenzyl carbonate; GAPDH: glyceraldehyde-3-phosphate dehydrogenase.

Additionally, GSH-depleting benzoyl-dibenzyl carbonate (B2C)-encapsulated small extracellular vesicles (BsEVs) have been engineered to bypass the drug efflux mechanisms and induce oxidative stress through GSH depletion, resulting in apoptosis in OVCAR-8 and OVCAR-8/multidrug-resistant (MDR) ovarian cancer cells. This approach has demonstrated significant anticancer efficacy against multidrug-resistant human ovarian cancer cells. BsEVs enhance drug sensitivity by inhibiting adenosine triphosphate (ATP) production, utilizing nicotinamide adenine dinucleotide (NADH), and inducing mitochondrial dysfunction. This disruption impairs the function of efflux pumps that typically contribute to drug resistance. *In vivo* studies have shown that treatment with BsEVs significantly inhibits the growth of OVCAR-8/MDR and OVCAR-8 tumors. Furthermore, evidence suggests that OVCAR-8/MDR tumors are more responsive to BsEV treatment compared to OVCAR-8 tumors. These findings highlight the promising potential of BsEVs in cancer therapy, particularly in overcoming multidrug resistance in ovarian cancer cells [98] (Fig. 8).

### 6.2 Exosomes improve delivery targeting

Researchers have explored the therapeutic potential of DDP loaded exosomes derived from M1 macrophages obtained from umbilical cord blood, particularly in the context of ovarian cancer and platinum resistance. Monocytes from umbilical cord blood can be differentiated into M1 or M2 macrophages through cytokine stimulation, and these macrophages can release exosomes encapsulating cisplatin. The use of M1-derived exosomes containing cisplatin (M1exoCIS) has been shown to significantly enhance the cytotoxic effects of the drug, restoring the sensitivity of ovarian cancer cells to cisplatin compared to traditional cisplatin formulations. The increased targeting and binding efficacy of M1exoCIS in ovarian cancer cells are largely due to its interaction with overexpressed integrin receptors. Exosomes from M1 macrophages contain lncH19, which leads to increased PTEN protein expression and heightened apoptosis in ovarian cancer cells upon treatment with M1exoCIS. Additionally, M1exoCIS suppresses the expression of miR-130a and *Pgp* genes in ovarian cancer cells, effectively overcoming cisplatin resistance [99].

To enhance the targeted delivery of exosomes to specific tissues or organs, exosome surface proteins can be modified, allowing for precise delivery to targeted lesions and enhancing therapeutic effects. In an *in vivo* xenograft model, researchers demonstrated that administering Arg-Gly-Asp (RGD)-modified exosomes loaded with miR-484 facilitated vascular normalization, thereby increasing the susceptibility of cancer cells to chemotherapy-induced apoptosis. Mechanistically, miR-484 suppressed the expression of vascular endothelial growth factor A (VEGF-A) and its receptors in endothelial cells. The targeted delivery of miR-484 via RGD-modified exosomes not only normalized vasculature but also enhanced the tumor’s sensitivity to chemotherapy [100].

Further research has examined the use of exosomes loaded with the Chuanxiong-derived alkaloid monomer, tetramethylpyrazine (TMP), known for its anti-tumor properties, including inhibition of tumor cell proliferation, invasion, and drug resistance. Exosomes carrying TMP (EXO-TMP) have shown promising anti-ovarian cancer effects, especially when combined with PTX. EXO-TMP was effective in overcoming cellular drug resistance by suppressing the expression of multidrug resistance protein 1 (MDR1), multidrug resistance-associated protein 1 (MRP1), and glutathione S-transferase Pi 1 (GSTP1). Moreover, EXO-TMP demonstrated specific targeting capabilities toward A2780T cells, effectively mitigating their drug resistance. These findings suggest that EXO-TMP could be a promising drug delivery strategy for reversing drug resistance in ovarian cancer [101].

### 6.3 Enhancing drug delivery with exosomes

Triptolide, a bioactive compound derived from the Chinese herbal remedy *Tripterygium wilfordii*, exhibits potent anti-tumor, immunomodulatory, and anti-inflammatory properties. However, its clinical use is limited by significant adverse effects. To address these limitations, researchers have developed exosome-encapsulated formulations of triptolide (TP-Exos). This innovative delivery system has demonstrated a marked improvement in the anti-cancer efficacy of triptolide against ovarian cancer while reducing its toxicity to vital organs compared to the unencapsulated drug. According to Guo et al. [102], TP-Exos induced apoptosis in ovarian cancer cells, modulated tumor immunity by activating the mitochondrial apoptotic pathway, and selectively suppressed M2-type tumor-associated macrophages and their pro-tumor factors in the tumor microenvironment. These findings suggest that TP-Exos could be a promising therapeutic option for ovarian cancer.

**Fig. 9:**
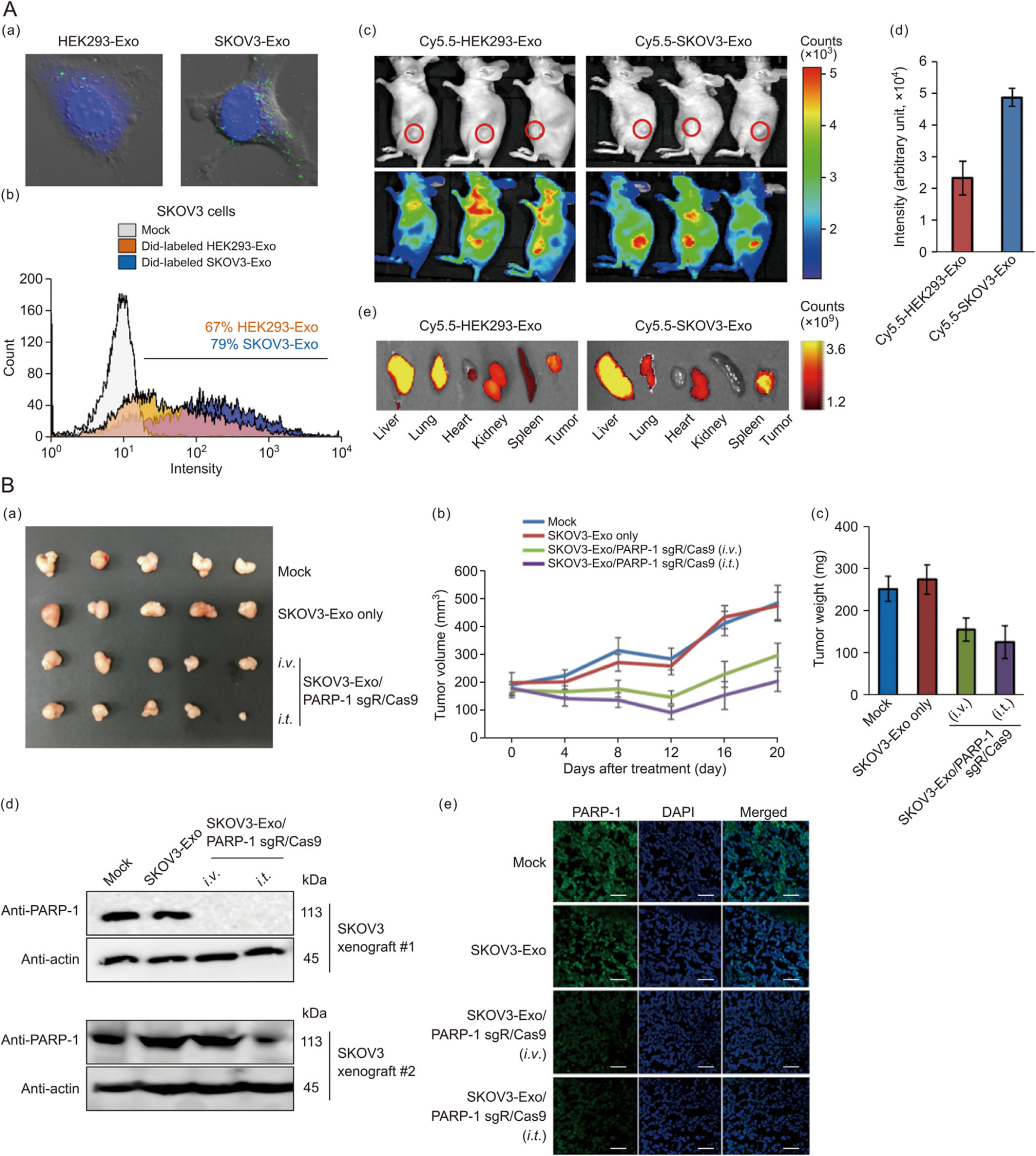
Exosomes serve as delivery vehicles for targeted cancer therapy. (A) Exosome-mediated *in vitro* cellular uptake and *in vivo* biodistribution. (a) Confocal images exhibited the cellular internalization of two types of exosomes that originated from HEK293 and SKOV3 in SKOV3 cancer cells *in vitro*. To visualize the exosomes, Dio (green), a lipophilic dye, was labeled. (b) Flow cytometry (FACS) analysis showed *in vitro* cellular uptake of the Did- (red), a lipophilic dye, labeled exosomes at 3 h post-treatment. (c) *In vivo* biodistribution of Cy5.5-labeled exosomes intravenously administered to SKOV3 xenograft mice. (d) Fluorescence intensity of Cy5.5-labeled exosome at tumors. (e) *Ex vivo* images were visualized using in vivo imaging system (IVIS) spectrum. Printed from Ref. [103] with permission. (B) Anti-cancer effects of cancer exosomes loaded with clustered regularly interspaced short palindromic repeats (CRISPR)/CRISPR-associated protein 9 (Cas9) in SKOV3 xenografts. CRISPR/Cas9-loaded exosomes were administered intravenously and intratumorally with an interval of 3 days two times. (a) Excised tumors on day 28 after the initial injection. (b) Tumor growth was measured from the day of injection. (c) Tumor weight of mice at the end point of experiments. (d, e) Levels of poly(ADP-ribose) polymerase 1 (PARP-1) were analyzed by western blotting analysis (d) and immunofluorescence staining (e) in excised tumors of xenografts. For western blotting, two individual tumors were used. Scale bars, 100 μm. Printed from Ref. [103] with permission. DAPI: 4',6-diamidino-2-phenylindole.

The efficacy of clustered regularly interspaced short palindromic repeats (CRISPR)/CRISPR-associated protein 9 (Cas9) as a genome-editing tool depends on efficient intracellular delivery systems. Recent studies have demonstrated that tumor cell-derived exosomes can effectively transport CRISPR/Cas9 plasmids to cancer tissues. Specifically, these exosomes exhibit superior delivery capabilities *in vivo* compared to those derived from epithelial cells, as they selectively accumulate in ovarian cancer tumors in SKOV3 xenograft mice, likely due to their cell-specific tropism. Exosomes carrying CRISPR/Cas9 have been shown to suppress poly (ADP-ribose) polymerase-1 (PARP-1) expression, inducing apoptosis in ovarian cancer cells. Furthermore, CRISPR/Cas9-mediated inhibition of PARP-1 enhances the sensitivity of these cells to cisplatin, leading to a synergistic cytotoxic effect [103] (Fig. 9).

N6-methyladenosine (m6A) regulators play crucial roles in the fate of m6A-modified transcripts, cancer progression, and drug resistance. Gene therapy using siRNA shows potential for correcting aberrant expression of m6A regulators, though challenges with specificity and systemic toxicity necessitate the use of targeted delivery vectors. Researchers have developed a dual-purpose nanomedicine platform combining regulation of m6A-related epigenetics with chemotherapy for ovarian cancer treatment. This system involves encapsulating siRNAs targeting YT521-B homology (YTH) N6-methyladenosine RNA-binding protein 1 (YTHDF1) and the chemotherapy docetaxel (DTX) within small extracellular vesicles derived from mesenchymal stem cells. This approach effectively targets tumors, depleting YTHDF1 and suppressing eukaryotic translation initiation factor 3 subunit C (EIF3C) translation in an m6A-dependent manner. The synergistic interaction between YTHDF1 targeting and epigenetic regulation significantly enhances the antitumor efficacy of DTX, suppressing ovarian cancer progression without notable systemic toxicity [104].

**Fig. 10:**
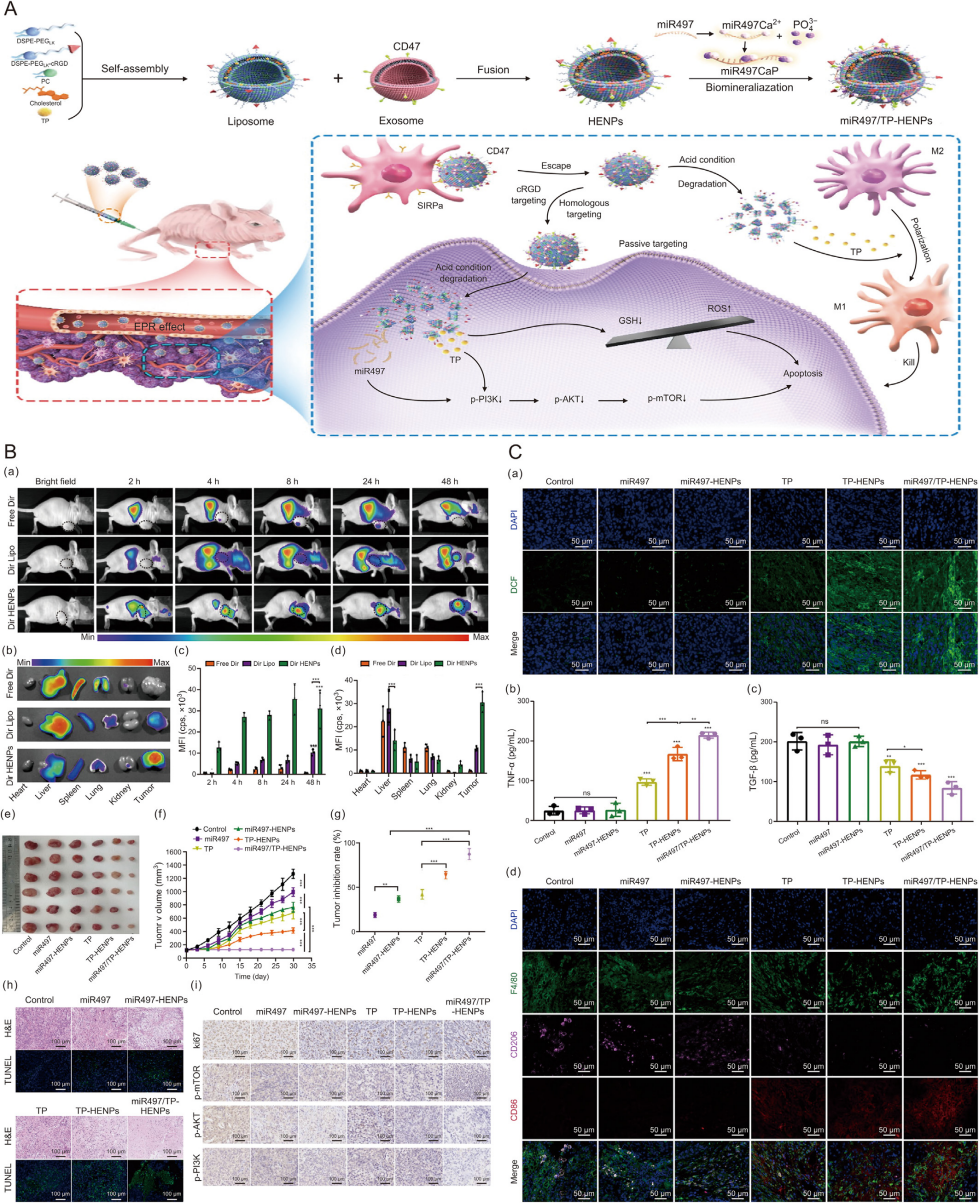
Nanoparticles deliver miR-497 to target chemoresistant ovarian cancer. (A) Diagram of the formative process and mechanism of action of miR497/tumor-targeting peptide-hybrid engineered nanoparticles (TP-HENPs). Printed from Ref. [105] with permission. (B) The targeting and antitumor activity of miR497/TP-HENPs *in vivo*. (a) *In vivo* imaging to observe the tumor targeting ability of different nanoparticles. (b) *Ex vivo* fluorescence images of the main organs and tumors isolated from mice bearing subcutaneous cisplatin-resistant SKOV-3 subline (SKOV3-CDDP) tumors. (c) Quantitative analysis of 1,1'-dioctadecyl-3,3,3',3'-tetramethylindotricarbocyanine iodide (Dir) distribution in the tumor site postinjection elevated by the fluorescence intensity measured in (a). (d) Quantitative assessment of the mean fluorescence intensity in major organs and isolated subcutaneous tumors. (e) Representative photographs of subcutaneous tumors harvested from all treatment groups. (f) Growth record curves of subcutaneous tumors in nude mice during the experiment. (g) The inhibition rate of ovarian cancer (OC) treated with various drugs. (h) The hematoxylin and eosin (HE) staining and TdT-mediated dUTP nick-end labeling (TUNEL) staining. (i) Immunohistochemical detection of Ki67, phosphorylated phosphatidylinositol 3-kinase (p-PI3K), phosphorylated protein kinase B (p-AKT), and phosphorylated mammalian target of rapamycin (p-mTOR). Printed from Ref. [105] with permission. (C) miR497/TP-HENPs induced reactive oxygen species (ROS) production in cisplatin-resistant ovarian cancer and regulated macrophage polarization from M2 to M1 in vivo. (a) The ROS of tumor sections after treatments. (b, c) Serum levels of Tumor necrosis factor alpha (TNF-α) and transforming growth factor beta 1 (TGF-β1) in different treatment groups *in vivo*. (d) Immunofluorescence staining images of different macrophage markers (M1, F4/80 + CD86+ and M2, F4/80 + CD206+) in tumor tissue sections. Printed from Ref. [105] with permission. DSPE-PEG: 1,2-distearoyl-sn-glycero-3-phosphoethanolamine-polyethylene glycol; PC: phosphatidylcholine; SIRPα: signal regulatory protein α; EPR: enhanced permeability and retention effect; GSH: glutathione; DCF: 2',7'-dichlorodihydrofluorescein diacetate.

Biomimetic hybrid nanoparticles, termed miR497/triptolide-loaded hybrid exosome-lipose nanoparticles (TP-HENPs), have been developed to co-deliver miR497 and triptolide. *In vitro* studies show that these nanoparticles are efficiently taken up by tumor cells, resulting in increased apoptosis. Moreover, they effectively accumulate at the tumor site and exhibit strong anti-cancer efficacy *in vivo* without adverse effects. Mechanistically, these nanoparticles facilitate the dephosphorylation of the hyperactivated PI3K/AKT/mammalian target of rapamycin (mTOR) signaling pathway, enhance the generation of reactive oxygen species (ROS), and promote the shift of macrophages from the M2 to M1 phenotype. The co-delivery of TP and miR497 using exosome-liposome hybrid nanoparticles has proven especially effective in overcoming chemoresistance in ovarian cancer, presenting a promising strategy for cancer therapy [105] (Fig. 10).

## 7 Surface-enhanced Raman scattering (SERS) detection of ovarian cancer exosomes

EVs, including exosomes, hold significant potential in cancer therapy due to their unique molecular characteristics [106]. However, the heterogeneity and complexity of small extracellular vesicles (sEVs) pose substantial challenges for accurate molecular profiling. Traditional methods such as Western blotting, ELISA, and flow cytometry, while useful, often fall short in terms of sensitivity, specificity, multiplexing capability, and the need for large sample volumes. Over the past few decades, various technologies have been developed to enhance the accuracy, specificity, and sensitivity of sEV detection, including chemiluminescence, electrochemistry, and notably, SERS.

SERS has emerged as a highly sensitive and rapid method for analyzing multiple proteins on the surface of sEVs. Compared to other sEV detection technologies, SERS offers advantages such as low noise [107,108], resistance to photobleaching, robustness, and high sensitivity. As an optical technique, SERS enhances Raman scattering signals when molecules are in close proximity to metal nanoparticles. Since its discovery in the 1970s, SERS has been applied across various fields, including drug development and food quality control, and has shown particular promise in the detection of sEVs for cancer diagnosis.

In sEV analysis, SERS-based immunoassays commonly utilize a “sandwich” format. In this setup, sEVs are captured using probes like antibody-coupled magnetic beads, and detection is achieved using SERS nanotags, which consist of Raman-active molecules encapsulated with metal nanoparticles. The resulting “sandwich” immunocomplex provides a platform for detecting sEVs [109]. There are three main strategies for assembling these immunocomplexes: labeling sEVs with SERS nanotags followed by enrichment with capture probes, simultaneous incubation of all components, or enrichment with capture probes prior to labeling with SERS nanotags. However, comprehensive studies comparing the efficacy of these different assembly strategies in SERS detection of sEVs are lacking. Such studies are essential for developing standardized protocols for using SERS in sEV detection.

The membrane of sEVs typically contains common biomarkers such as CD9, CD63, and CD81, which are part of the tetraspanin family. These biomarkers are frequently used to capture sEVs and form the “sandwich” immunocomplex for detection. However, tetraspanins are not uniformly distributed on the surface of sEVs, and their expression levels may vary across different types of cancer-derived sEVs. Most studies have relied on anti-CD63 antibodies to capture sEVs from various cancer types, potentially missing a significant portion of sEVs due to the variable expression of this protein. Identifying a more abundant tetraspanin protein specific to ovarian cancer-derived sEVs (OVC-derived sEVs) could significantly improve the capture efficiency and sensitivity of the assay. This advancement would enhance the overall accuracy of sEV-based diagnostics and facilitate the detection of ovarian cancer [110,111].

Recent research has focused on identifying specific surface proteins to classify and characterize sEV phenotypes, particularly for detecting ovarian cancer. Surface markers such as cancer antigen 125 (CA125), EpCAM, and CD24 have been selected from various cell lines for this purpose [112]. A notable approach involves using gold nanopore arrays to capture ovarian cancer-derived EVs, which are then characterized using SERS. This method, combined with principal component analysis (PCA), allows for the identification of distinct component differences among the EVs. A logistic regression-based machine learning model was subsequently applied, achieving approximately 99% accuracy, sensitivity, and specificity in classifying the EVs [113]. This method is not only quick and easy but also non-invasive, making it a highly practical diagnostic tool.

In another study, a simple platform utilizing plasma brackets embedded with microscale biological silicate substrates and silver nanoparticles was developed for SERS analysis of EVs from ovarian and endometrial carcinomas. The sensitivity of detecting these cancers was found to decrease significantly after enzymatic digestion of the EVs, highlighting the critical role of intact EVs in diagnostic accuracy. This platform underscores the importance of using SERS as a tool for evaluating the heterogeneity of EVs from clinical samples in a cost-effective, rapid, and label-free manner [114].

Further advancements in SERS technology, combined with mass spectrometry (MS), have been used to analyze EVs isolated from plasma samples of healthy donors (HD), umbilical cord blood, and patients with early-stage high-grade serous carcinoma (HGSC). Plasma EVs were purified using size exclusion chromatography and subsequently analyzed with SERS, MS, and atomic force microscopy. After identifying the fraction with the highest EV purity, the study characterized EVs from HDs, donors with non-cancerous gynecological conditions, and early-stage HGSC patients. These advanced analytical techniques allow for detailed profiling and comparison of EVs, providing valuable insights into the molecular composition and potential biomarkers associated with different stages of ovarian cancer.

This comprehensive approach using SERS and complementary techniques demonstrates the potential for developing highly sensitive and specific diagnostic tools for ovarian cancer. The integration of SERS with machine learning and other analytical technologies offers a powerful means to enhance the accuracy of cancer diagnostics, particularly in the context of EV characterization [115].

## 8 Challenges and prospects

Exosomes have shown significant potential in tumor biology, particularly as carriers of tumor-specific antigens and nucleic acids. These features enable exosomes to serve as valuable diagnostic and prognostic biomarkers for noninvasive cancer assessment. Moreover, exosomes can help identify individuals at risk of metastatic disease, and manipulating their production may present novel therapeutic targets. The use of exosomes as vehicles for delivering cellular information offers a promising approach for targeted cancer therapy, with functional exosome mimetics potentially enhancing drug efficacy [116]. However, while various synthetic drug delivery systems have been developed, their clinical application has often been limited by challenges such as inefficiency, cytotoxicity, and immunogenicity [117]. The negative charge on the surface of exosomes contributes to their stability in the bloodstream and facilitates the efficient delivery of biomolecules to target cells, making them effective drug carriers [118].

Despite the growing interest in exosome research, several unresolved questions remain. Most exosome-based treatments are still in the experimental phase and lack extensive clinical trials. One major challenge is the isolation of exosomes, which involves addressing issues related to standardization of isolation methods, exosome heterogeneity, potential toxicity of therapeutic exosomes, and detailed investigation into their molecular components [119]. The complexity of exosome heterogeneity stems from factors such as size, molecular diversity, and varying cellular origins [120]. In ovarian cancer patients, circulating exosomes are a mixture of vesicles released from different regions of the female reproductive system. To effectively tackle these complexities, a unified approach to exosome analysis, encompassing both isolation and characterization, has been suggested. Techniques like microfluidic devices, magnetic bead-based methodologies, and aptamer-based separation can be employed for precise exosome isolation [[121], [122], [123], [124]].

For the molecular analysis of exosomal cargo, a range of advanced techniques are available. These include nanopore-based detection of individual molecular signals, CRISPR-based sensors for analyzing surface proteins specific to cancer, and plasma sensors for categorizing distinct exosome subsets. Additionally, electrochemical sensors can provide valuable insights. The integration of multi-omics approaches—genomics, transcriptomics, and proteomics—coupled with machine learning, enables the identification of specific cancer biomarkers at the final stage of individual exosome analysis. Such comprehensive analysis is critical for advancing precision and personalized medicine in cancer treatment. A crucial aspect of exosome research involves the toxicological evaluation of exosomes, as the secretion patterns and molecular cargo of exosomes can influence the behavior of recipient cells, particularly when exposed to various substances, including drugs and chemicals [125].

Recent studies have highlighted the non-toxic nature of mesenchymal stem cell (MSC)-derived exosomes. However, it is also important to explore the therapeutic potential of exosomes from other sources, such as plant-derived exosomes and dendritic cell-derived exosomes. Comprehensive toxicological analyses of these alternative exosome sources are necessary to fully understand their implications and safety profiles. Further research in this area will help clarify the potential risks and benefits, paving the way for the safe and effective use of exosomes in clinical applications [126,127].

Emerging evidence underscores the pivotal role of exosomes in mediating communication between cancer cells and the immune system. Targeting tumor-derived exosomes offers a promising avenue for reducing immunosuppression and enhancing the efficacy of immunotherapy [128]. However, before exosomes can be utilized in clinical immunotherapy for ovarian cancer, it is essential to thoroughly evaluate their potential side effects to optimize their therapeutic effectiveness. Overcoming these challenges could usher in a new era of exosome-based cancer vaccines, while exosome research continues to advance the realization of precision medicine.

The reproducibility, reliability, and sensitivity of SERS-based biosensing technology depend on the rational design of SERS substrates, proper handling of biological samples, precise control of detection conditions, and thorough analysis of spectral data. Efforts should be made to control potential influencing factors in the future. SERS substrates with high uniformity, strong enhancement effects, and clean surfaces should be fabricated and appropriately modified. Choosing suitable Raman probe molecules can also enhance biocompatibility and avoid non-specific adsorption during testing. Additionally, biological samples should be stored and pretreated as closely as possible to normal physiological conditions to prevent irreversible changes. Selecting appropriate SERS detection conditions is vital for obtaining reliable detection information. Moreover, it is essential to establish a standardized biomolecule SERS database for accurate spectral information identification. If necessary, calibration of the probe responses in the detection system should be performed, or suitable data processing and statistical analysis methods should be employed. The future of early detection of tumor biomarkers lies in the development of ultra-sensitive, highly specific, high-throughput, and universal detection platforms to meet the needs of life sciences and clinical diagnosis. While the application of SERS technology has made significant strides, numerous challenges remain in accelerating the translation of SERS technology. Collaborative approaches involving nanotechnology, materials science, plasmonics, and optics will continuously enhance exosome research.

Modifying the surface molecules of exosomes to achieve cell and tissue targeting specificity represents a potent strategy for precise delivery of therapeutic agents. By loading proteins, small molecules, or functional genetic material into exosomes, it is possible to direct therapeutic effects specifically to diseased areas or target cells. Exosomes have the potential to function as nanocapsules for the delivery of chemotherapeutic drugs, particularly in tackling drug-resistant tumors. However, thorough investigation, validation, and standardized clinical trials are crucial to confirm the safety and efficacy of exosome-based therapies before their widespread clinical use.

While exosomes offer numerous advantages as drug carriers, such as biocompatibility and ability to bypass certain drug resistance mechanisms, they also face challenges including limited drug-loading capacity, production and purification difficulties, and vulnerability to degradation during storage and transportation. Successfully incorporating nucleic acid drugs into exosomes, identifying suitable host cells for their production, and ensuring stable ligand-receptor interactions for targeted delivery remain critical hurdles for clinical applications. Optimizing the combination of producer and target cells is essential for efficient therapeutic exosome production.

This field, characterized by its potential for innovation, continues to evolve rapidly. With ongoing research and technological advancements, new strategies are likely to emerge, enhancing the use of exosomes in cancer diagnosis and treatment, thus significantly impacting patient care.

## 9 Conclusions

The significant role of exosomes in tumor drug resistance and their potential as efficient carriers are increasingly recognized. By harnessing the unique properties and intercellular communication capabilities of exosomes, researchers have gained critical insights into the complex mechanisms underlying cancer progression, metastasis, and drug resistance. Although challenges persist regarding exosome heterogeneity, isolation techniques, potential toxicity, and comprehensive molecular analysis, ongoing advancements in knowledge and technology are steadily overcoming these obstacles.

Current research is exploring the utility of exosomes as a valuable tool in combating ovarian cancer, particularly in enhancing drug delivery systems, overcoming drug resistance, and ultimately improving survival outcomes. This exploration is anticipated to lead to significant advancements in precision and personalized medicine for ovarian cancer treatment. Additionally, the application of SERS technology in studying exosomes presents a promising approach for understanding drug resistance in ovarian cancer. As this field evolves, it is poised to revolutionize diagnostic and therapeutic strategies, ushering in a new era of tailored cancer care.

## CRediT authorship contribution statement

**Biqing Chen:** Writing – original draft. **Xiaohong Qiu:** Writing – review & editing. **Yang Li:** Writing – review & editing.

## Declaration of competing interest

The authors declare that there are no conflicts of interest.
